# Sulfated Glycosaminoglycans as Inhibitors for Chlamydia Infections: Molecular Weight and Sulfation Dependence

**DOI:** 10.1002/mabi.202400443

**Published:** 2025-01-21

**Authors:** Sebastian Wintgens, Janita Müller, Felicitas Drees, Dominik Spona, Lorand Bonda, Laura Hartmann, Johannes H. Hegemann, Stephan Schmidt

**Affiliations:** ^1^ Heinrich‐ Heine‐ University Düsseldorf Faculty of Mathematics and Natural Sciences Institute for Functional Microbial Genomics 40204 Düsseldorf Germany; ^2^ Heinrich‐ Heine‐ University Düsseldorf Faculty of Mathematics and Natural Sciences Institute of Organic Chemistry and Macromolecular Chemistry 40204 Düsseldorf Germany; ^3^ Institute for Macromolecular Chemistry Faculty of Chemistry and Pharmacy Albert‐Ludwigs‐Universität Freiburg 79104 Freiburg Germany

**Keywords:** carbohydrate interaction, carrageenan, chlamydia pneumoniae, chondroitin sulfate, GAGs, glycans, heparan sulfate, heparin, sulfation

## Abstract

Glycosaminoglycans (GAGs) play a pivotal role in pathogen attachment and entry into host cells, where the interaction with GAGs is critical for a diverse range of bacteria and viruses. This study focuses on elucidating the specific interactions between sulfated GAGs and the adhesin OmcB (Outer membrane complex protein B) of *Chlamydia* species, examining how structural characteristics of GAGs, such as sulfation degree and molecular weight, influence their binding affinity and thereby affect bacterial infectivity. A surface‐based binding assay is established to determine the binding constants of OmcB with various GAGs. It is shown that increased sulfation and higher molecular weight enhance GAG binding to OmcB. These findings are further validated using cell assays, which shows that the addition of sulfated GAGs reduces OmcB‐cell binding and inhibits the attachment of *C. pneumoniae* elementary bodies (EBs), underscoring the pivotal role of specific GAGs in chlamydial infections. Notably, heparin exhibites a stronger inhibitory effect on OmcB compare to GAGs with similar sulfation degrees and molecular weights, suggesting that particular molecular architectures may optimize binding interactions.

## Graphical abstract

Interactions between sulfated glycosaminoglycans (GAGs) and the adhesin OmcB of Chlamydia species are explored using a surface‐based binding assay and cell assays. Increased sulfation and molecular weight enhance GAG binding and reduce bacterial attachment, with heparin exhibiting superior inhibitory effects. The study highlights the critical role of GAG structure in pathogen infections.

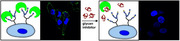

## 1 Introduction

Glycosaminoglycans (GAGs) are well known for facilitating pathogen attachment to host cells, a crucial step preceding entry of a variety of different cell types, or colonization and/or biofilm formation.^[^ ^1^ ^]^ GAGs are distributed throughout various cellular domains, including intracellular compartments, the cell glycocalyx, and the extracellular environment. There are four major classes of glycosaminoglycans (GAGs), distinguished by their core disaccharide structures. These include heparan sulfate (HS) and heparin, alongside the chondroitin sulfate/dermatan sulfate, keratan sulfate, and hyaluronan families, all of which play key roles in regulating essential cellular processes.^[^ ^2^, ^3^ ^]^ Heparin is distinct from HS in that it is produced primarily by mast cells, whereas HS is produced by all cell types. Moreover, heparin has a higher degree of sulfation compared to HS. GAGs are linear polysaccharides of highly complex structure, largely due to variable modification of their disaccharide subunits, which undergo *N*‐ and *O*‐sulfation, acetylation, and carboxylation. All GAG classes except the hyaluronan family are covalently attached to proteins (proteoglycans).

GAGs are found the in the outer cell layer, known as the glycocalyx, where they are displayed on proteoglycans, facilitating pathogen attachment. A recent example is SARS‐CoV‐2 that has evolved to show particularly strong binding to sulfated GAGs.^[^ ^4^ ^]^ Likewise, bacteria such as *P. aeruginosa*, *N. gonorrhoeae*, *Y. enterocolitica*, and S. aureus have been shown to exploit GAGs as a gateway for entering host cells, highlighting the diversity in bacterial exploitation of host GAGs for infection.^[^ ^5^, ^6^, ^7^, ^8^ ^]^ Other clinically relevant examples are Chlamydia, respiratory (C. pneumoniae) or urogenital tract and eye (*C. trachomatis*) pathogens, known to mediate first attachment to host cells via sulfated GAGs.^[^ ^9^, ^10^, ^11^ ^]^ The infection cycle of *Chlamydia* begins with the adhesion of the infectious elementary bodies (EBs) to the human cell and their subsequent internalization, using multiple adhesins on the EB surface for this interaction. The protein OmcB is present in the EBs of all chlamydial species, and was identified as a major component of the chlamydial outer membrane complex. The initial contact to host cells is established via OmcB by interacting with heparan sulfate‐like GAGs on the plasma membrane of the host cell.^[^ ^9^, ^10^ ^]^ This initial attachment is reversible, allowing the bacteria to surf the host cell surface in order to find a specific domain for cell entry with GAGs acting as a bridge element between the chlamydial and eukaryotic cell surface structures. It was shown that the adhesion to target cells is mediated by a region in the *N*‐terminal part of OmcB called the OmcB binding domain (OmcB‐BD).^[^ ^9^ ^]^ The interaction to sulfated GAGs is primarily due to two XBBXBX heparin‐binding motifs in OmcB‐BD, where X represents a non‐polar amino acid and B a cationic (basic) amino acid. Additionally, basic and polar amino acid residues located further C‐terminal to the XBBXBX motifs play a role in the binding of GAGs.^[^ ^9^ ^]^ The heparin‐binding motifs are predicted to fold in an α‐helix, while the random coil structure C‐terminal to the heparin‐binding motif contributes to the binding characteristics of OmcB to sulfated GAGs through ionic interactions and hydrogen bonding.^[^ ^12^ ^]^

Recent studies have elucidated that this interaction is not only pivotal for the initial attachment but also for the subsequent invasion of Chlamydia.^[^ ^9^, ^12^, ^13^ ^]^ Generally, the interaction between sulfated GAGs and pathogens is not limited to nonspecific ionic interaction; rather, it often involves specific motifs on bacterial surfaces that bind, depending on the sulfation pattern, the 3D structure of the glycosyl units, or the molecular weight and flexibility of the GAG.^[^ ^14^, ^15^, ^16^ ^]^ Looking at the broader picture of the role of GAGs during infection, GAGs are not just employed for pathogen attachment, rather pathogens may subvert GAGs on multiple stages of pathogenesis, including signaling, shedding of virulence factors, or disarming the hosts’ defense.^[^ ^1^, ^17^, ^18^ ^]^

The recognition of GAGs' role and glycan in general for pathogen adhesion has opened avenues for novel therapeutic interventions that target the pathogen attachment to the cell.^[^ ^19^, ^20^ ^]^ By designing multivalent carbohydrates that mimic the involved glycans and impeding pathogen binding, researchers aim to block the initial step of infection. The overall idea is that large multivalent molecules occupy the glycan binding sites of the pathogens (on the surface of the bacterial cell or the virion), thus impeding their attachment to the cells’ glycocalyx. Numerous different architectures have been proposed,^[^ ^21^, ^22^, ^23^ ^]^ for example polymers,^[^ ^24^, ^25^, ^26^, ^27^, ^28^, ^29^ ^]^ glycopeptides,^[^ ^30^ ^]^ glycodendrimers,^[^ ^31^, ^32^ ^]^ nanomaterials^[^ ^33^, ^34^, ^35^ ^]^ or gels.^[^ ^28^, ^36^, ^37^, ^38^, ^39^, ^40^ ^]^ As anti‐infection agents, these structures offer a promising approach, especially in the context of rising antibiotic resistance. However, the challenge lies in developing mimetics that are highly specific and efficient in disrupting pathogen‐glycan interactions without interfering with the host's physiological processes. For example, sulfated GAGs and their mimetics may show anticoagulant and antithrombotic activity,^[^ ^41^, ^42^ ^]^ or other unwanted side effects.^[^ ^43^ ^]^ This shows that GAGs with high specific binding to pathogens are needed, with a particular focus on understanding the roles played by GAG structural configurations, molecular weight, and the patterns and degrees of sulfation. In this context, the current study employs C. pneumoniae (Cpn) as a pertinent model pathogen, to explore how a spectrum of both natural and synthetic sulfated GAGs with varying sulfation degree and molecular weights inhibits the adhesin OmcB and bacterial infectivity. The *C*pn OmcB is unique among all chlamydial species as its heparan sulfate binding domain consists of at least 60 amino acids and includes two tandem heparin binding motifs, making it one of the largest GAG binding sites known in literature. Prior studies on the interaction between *Chlamydia* and GAGs were limited in scope, focusing on single or few GAG types, and while they confirmed the infection‐inhibiting properties of GAGs, they did not effectively quantify the inhibitors' relative strengths or fully explore the implications of sulfation degree and molecular weight.^[^ ^44^, ^45^, ^46^, ^47^, ^48^, ^49^ ^]^ Consequently, this work establishes a comprehensive screening assay to evaluate OmcB‐GAG interactions at a higher throughput, corroborates these findings through cellular assays, and further investigates the effect of these GAGs on the infectivity of *Cpn* EBs in cell cultures.

## 2 Results and Discussion

### 2.1 Glycan Screening Assay

**Figure 1:**
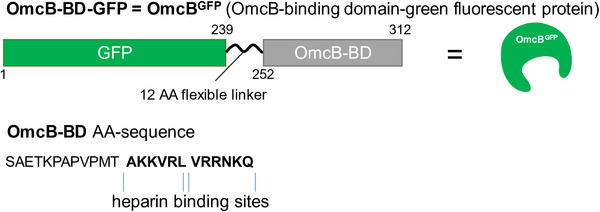
The GFP‐OmcB‐BD construct (OmcB^GFP^). The OmcB‐BD domain was N‐terminally tagged with a with GFP tag. The amino acid sequence (AA) represents OmcB‐BD without GFP. The heparin binding domain is highlighted in bold letters.

**Figure 2:**
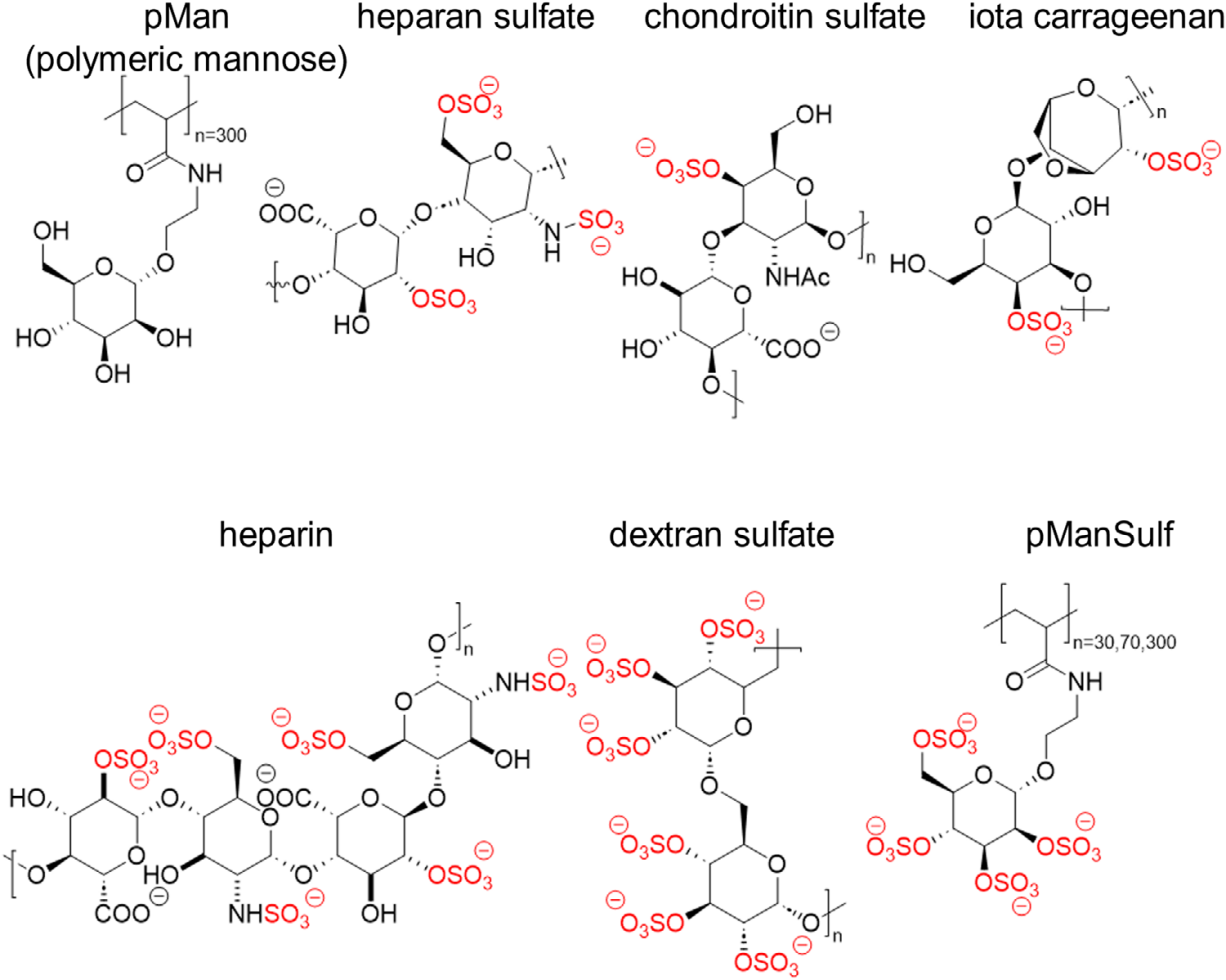
The series of artifical and natural glycans with varying sulfation degree and molecular weight (see **Table** **1**) to be used for the binding assays. Note that heparan sulfate is particularly structurally diverse due to varying degrees of sulfation and epimerization.

As a starting point, we developed a screening assay to assess the binding of a range of sulfated glycans using a well‐plate readout. For the readout, the GAG binding domain of the *Cpn* outer membrane protein (OmcB‐BD) was used, which is located between amino acid position 41 and 100. The OmcB.BD harbors two tandem XBBXBX motifs at position 53 to 64, showing the typical pattern of non‐polar and cationic amino acids (**Figure** **1**). The OmcB‐BD fragment was N‐terminally tagged with GFP (green fluorescent protein) to allow for fast readout by a microplate reader and optical microscopy (from here on referred to as OmcB^GFP^). The recombinant preparation and purification of OmcB^GFP^ was performed as previously described.^[^ ^12^ ^]^ A series of synthetic and natural sulfated glycans was established to be used in the binding assay (structures, **Figure** **2**). The degree of sulfation was measured by elemental analysis (Table S1, Supporting Information). In addition, sulfated polymannose structures (pManSulf) were synthesized by free radical photopolymerization (Figures S4–S9, Supporting Information). As a non‐sulfated control, a polymeric mannose (pMan) polymer was prepared without sulfation.

**Figure 3:**
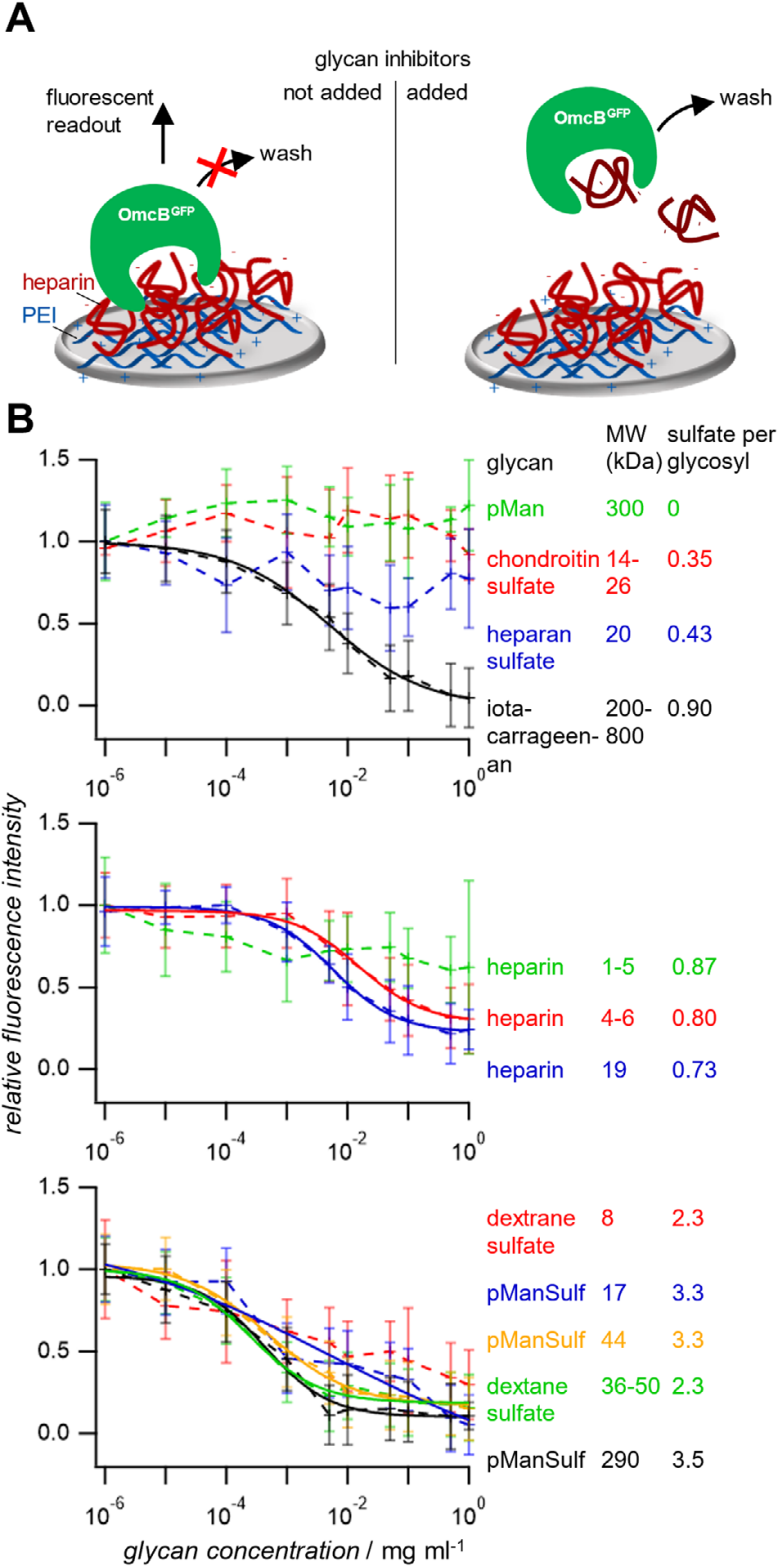
OmcB‐heparin inhibition competition assay. A) schematic representation of the assay. B) relative fluorescence intensity as a function of the glycan concentration, represented as data‐points connected by a dashed line. Not all data could be fitted by the Hill‐equation. The solid superimposed lines represent the fits. The values represent averages from triplicate measurement and the error bars represent the standard deviation.

The relative affinity of the GAGs to the recombinant OmcB^GFP^ was determined by a competition inhibition assay. As a reference surface for OmcB binding, we prepared a coating composed of branched polyethyleneimine (PEI) and heparin on glass bottom microplates. The polycation PEI was physisorbed on the glass surfaces followed by physisorption of the polyanionic heparin (19 kDa). This type of polyelectrolyte bilayer shows a negative surface charge as determined by colloidal probe adhesion measurements^[^ ^50^ ^]^ and specific adhesion of OmcB^GFP^ as shown by initial tests using optical microscopy (Figure S1, Supporting Information), i.e., the heparin layer is accessible for OmcB binding. Furthermore, the polyelectrolyte buildup was validated by quartz crystal microbalance measurements (Figure S2, Supporting Information). For the competition inhibition assay, the heparin coated microwells were filled with OmcB^GFP^ solutions (0.2 mg ml^−1^) containing the different glycan structures at different concentrations (up to 1 mg ml^−1^). After equilibration for 30 min, the GFP fluorescence signals were read out and plotted against the glycan concentration (**Figure** **3**). The inhibitory concentration at half maximum IC_50_ was determined as measure of the relative binding strength of the different glycans.

From the inhibition measurements, two main conclusions could be drawn. 1) An increased degree of sulfation generally leads to lower IC_50_ values, i.e., a stronger binding of the glycan. 2) Increasing the molecular weight of the glycan leads to stronger binding as well. The effect of sulfation can be readily seen by comparing the curves in Figure 3 that were grouped according to the number of sulfate groups per glycosyl. Only samples with no sulfate groups (pMan) or low sulfation degree (chondroitin sulfate, heparan sulfate) showed inhibition smaller that 50% at a concentration of 1 mg ml^−1^. The strongest inhibitors (dextran sulfates, pManSulf) had more than two sulfates per glycosyl unit. The effect of molecular weight is best seen when comparing the three heparin samples, showing progressively stronger inhibition at increased chain length. These results suggest that the inhibitory potential of the GAGs is largely governed by a polyelectrolyte effect, i.e., a higher charge density leads to strong binding to the cationic OmcB. The effect of the molecular weight could be explained by a steric shielding effect. Large molecules may efficiently block the binding site even when just weakly associating to the protein. In addition, statistical (re‐)binding is possible for the multivalent polyelectrolyte‐like molecules investigated here, i.e., statistically there is a higher propensity that rebinding of larger multivalent molecules occurs after dissociating from the protein.

The assay also indicates specific binding events apart from pure ionic interactions in case of heparin. For example, heparin at a molecular weight of 19 kDa and one sulfate per glycosyl showed a similar inhibitory potential when compared to pManSulf at a molecular weight of 20 kDa and 3.3 sulfate groups per glycosyl. Moreover, low molecular weight dextran sulfate sample showed clearly weaker inhibition compared to heparin despite having more than twice the sulfate units per glycosyl. Heparin appears to possess a relatively high affinity, considering the low molecular weight and degree of sulfation of the samples tested here. This suggests specific binding at the heparin‐binding motif of OmcB, as predicted by molecular modelling.^[^ ^51^ ^]^ Furthermore, unexpectedly, heparan sulfate exhibited only weak inhibition, although it is widely assumed to be the natural ligand of OmcB. A possible explanation for this could be that Chlamydia need to attach to heparan sulfates in the glycocalyx only weakly to be able to migrate and bind to entry receptors at the membrane for successful infection. Additionally, the degree of sulfation in heparan sulfate, known to have areas of high sulfation, might result in fewer high‐affinity binding sites. Further investigation is required to confirm these hypotheses.

Overall, the developed screening assay offers a versatile platform for assessing the binding affinity of sulfated glycans, leveraging the specific interaction between OmcB^GFP^ and heparin. The inclusion of a GFP tag facilitated efficient readout, enabling rapid assessment via microplate reader and optical microscopy. This approach not only provides insights into the binding preferences of OmcB^GFP^ but also serves as a valuable tool for investigating interactions between other sulfated glycans and their respective binding partners.

The utilization of synthetic and natural sulfated glycans, along with a non‐sulfated control, allows for comprehensive exploration of structure‐activity relationships governing glycan‐protein interactions. The determination of sulfation degree and molecular weight ensures precise characterization of the glycan samples, essential for accurate evaluation of binding affinity.

### 2.2 Cell Assays

#### 2.2.1 OmcB Binding to Different Cell Lines and Inhibition Studies

After conducting the screening studies on artificial heparin surfaces, we sought to validate the inhibition assay with different cell lines presenting natural GAG surfaces as OmcB binding sites. The two main cell lines tested were HEp‐2 and CHO‐WT (wild type Chinese hamster ovary cells). Both are widely used in cell biological research, and present heparan sulfate and chondroitin sulfate as major components of their glycocalyx.^[^ ^52^, ^53^ ^]^ Particularly, wild‐type and GAG‐defective CHO cells have been used to study how altering the GAG structure of proteoglycans affects fundamental properties of cells.^[^ ^53^ ^]^ Here we used two mutants, the glucosaminoglycan‐negative pgsA‐745 and the heparan sulfate (HS)‐negative pgsD‐677 cell line.

**Figure 4:**
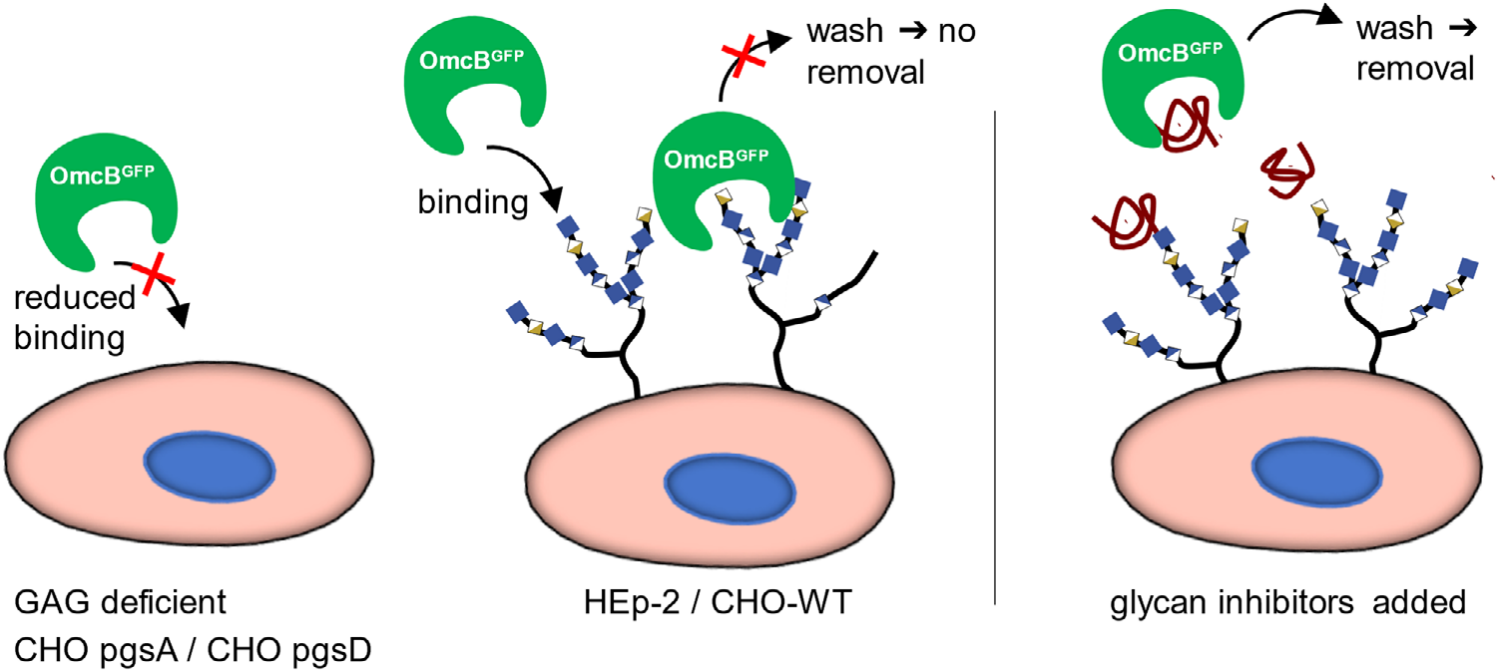
Sketch of the OmcB binding inhibition assay using various cells.

**Figure 5:**
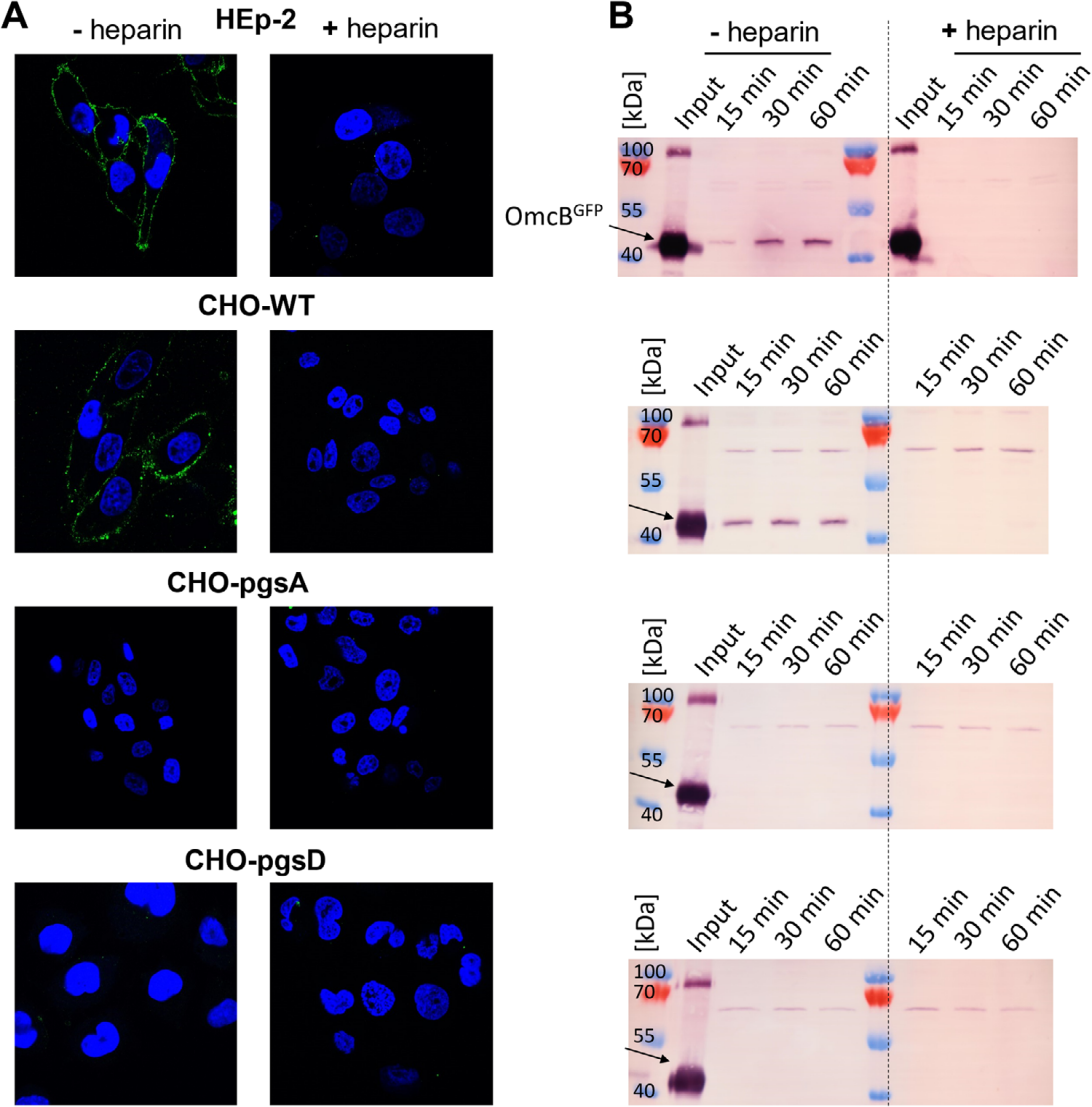
OmcB^GFP^ binding to different cell lines either presenting endogenous levels of sulfated GAGs, HEp‐2 and CHO‐WT (top two lanes), or presenting low levels of sulfated GAGs, CHO‐pgsA and CHO‐pgsD (bottom two lanes). A) Confocal microscopy studies showing the nucleus (blue, DAPI staining) and the cell surface in case OmcB binds (green, GFP channel). Only HEp‐2 and CHO‐WT show OmcB binding. Presence of 0.5 mg ml^−1^ heparin hinders binding. B) Western blots of cells lysed by phospho‐lysis buffer confirm binding of OmcB exclusively to HEp‐2 and CHO‐WT in absence of heparin. The dashed line is a guide to the eye separating gels with and without added heparin (see Figures S12–S15 (Supporting Information) for control assays).

Binding was studied via western blot and confocal microscopy, see **Figure** **4** for a sketch. For the binding studies, OmcB^GFP^ was added to adhered cell layers of the different cell lines. This was done either with or without pre‐incubation with heparin (0.5 mg ml^−1^). Cells were detached by incubation with cell‐dissociation solution, transferred to a reaction tube, and pelleted by centrifugation. The cell dissociation solution was then removed and the pellet was resuspended in PBS, protein blue marker and DTT. After incubation, all samples were analyzed on Western blots. As expected OmcB^GFP^ bound to HEp‐2 and CHO‐WT due to the presence of sulfated GAGs. Under incubation with heparin, binding did not take place. CHO pgsA and pgsD cells did not bind OmcB^GFP^ at all (**Figure** **5**). This clearly shows that the sulfated GAG species on HEp‐2 and CHO‐WT cells specifically bound OmcB^GFP^ and that the interaction could be inhibited by the competitive binder heparin at concentrations larger than the IC_50_ as measured by the screening assay (Table 1).

**Table 1:** Overview of the different glycans and their inhibitory potential with respect to OmcB binding. The molecular weights represent values given by the manufacturer, with the exception of pMan‐samples, which were determined by GPC. The sulfate groups were quantified by elemental analysis (Table S1, Supporting Information).

Glycan	MW / kDa_−1_	Sulfate groups per glycosyl	IC_50_ / µg mL^−1^	Inhibition at 1 mg mL
pMan83	83	0	No fit	no inhibition
Heparan sulfate	20	0.43 ± 0.05	No fit	no inhibition
Hondroitin sulfate	14–26	0.35 ± 0.05	No fit	no inhibition
Iota‐carrageenan	200–800	0.90 ± 0.08	4.9 ± 1.1	90%
Heparin	1–5	0.87 ± 0.09	No fit	35%
Heparin	4–6	0.80 ± 0.08	15 ± 4	60%
Heparin	19	0.73 ± 0.07	5.8 ± 0.8	75%
pManSulf20	20	3.3 ± 0.40	5.9 ± 2.4	80%
pManSulf42	42	3.3 ± 0.29	0.49 ± 0.13	80%
pManSulf198	198	3.5 ± 0.52	0.42 ± 0.15	90%
Dextran sulfate	8	1.34 ± 0.05	No fit	70%
Dextran sulfate	36–50	1.51 ± 0.05	0.24 ± 0.06	80%

**Figure 6:**
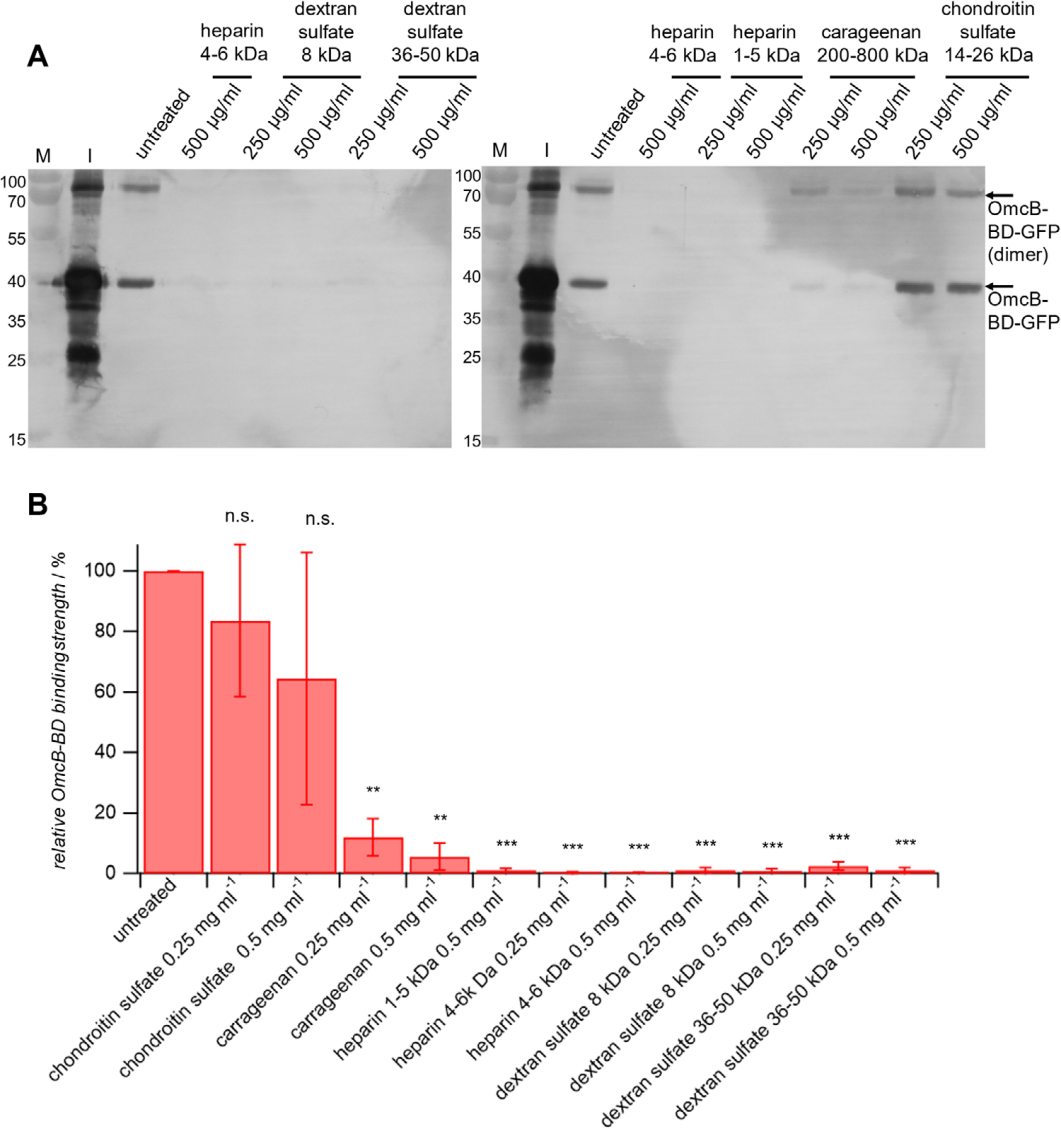
Inhibition of OmcB^GFP^ binding on HEp‐2 cells due to the presence of sulfated GAGs. A) Exemplary western blots. B) OmcB^GFP^ binding relative to the untreated control. Measurements for each condition were conducted three times. Data are presented as averages and the statistical significance was evaluated using *t*‐test (*<0.05, **<0.01, ***<0.001, ****<0.0001; ns: not significant).

To study the inhibitory potential of different glycans directly on cells, OmcB^GFP^ was incubated with the glycans at 250 µg ml^−1^ or 500 µg ml^−1^ and then added to HEp‐2 cells for one hour. As a readout, the amount of bound OmcB^GFP^ was determined via western blot as described above (**Figure** **6**). The results were in line with the screening assay using heparin coated microplates surfaces. Glycans with 0.5 sulfate group per glycosyl such as chondroitin sulfate and carrageenan showed low inhibition. Heparin with one sulfate group per glycosyl exhibited complete inhibition, similar to dextran sulfate with higher sulfate density. This is expected because the concentration of the dextran sulfates and heparin 4–6 kDa was considerably higher in this assay than their IC_50_ (Table 1). Heparin 1–5 kDa also showed complete inhibition of OmcB binding at 0.5 mg ml^−1^, which was not the case on heparin coated microplate surfaces. This could be explained by a much higher density of OmcB binding partners on the densely coated glass surfaces when compared to HEp‐2 glycocalyx. Therefore, the amount of inhibitor required was much lower in this cell‐based assay.

#### 2.2.2 Infection Assay

**Figure 7:**
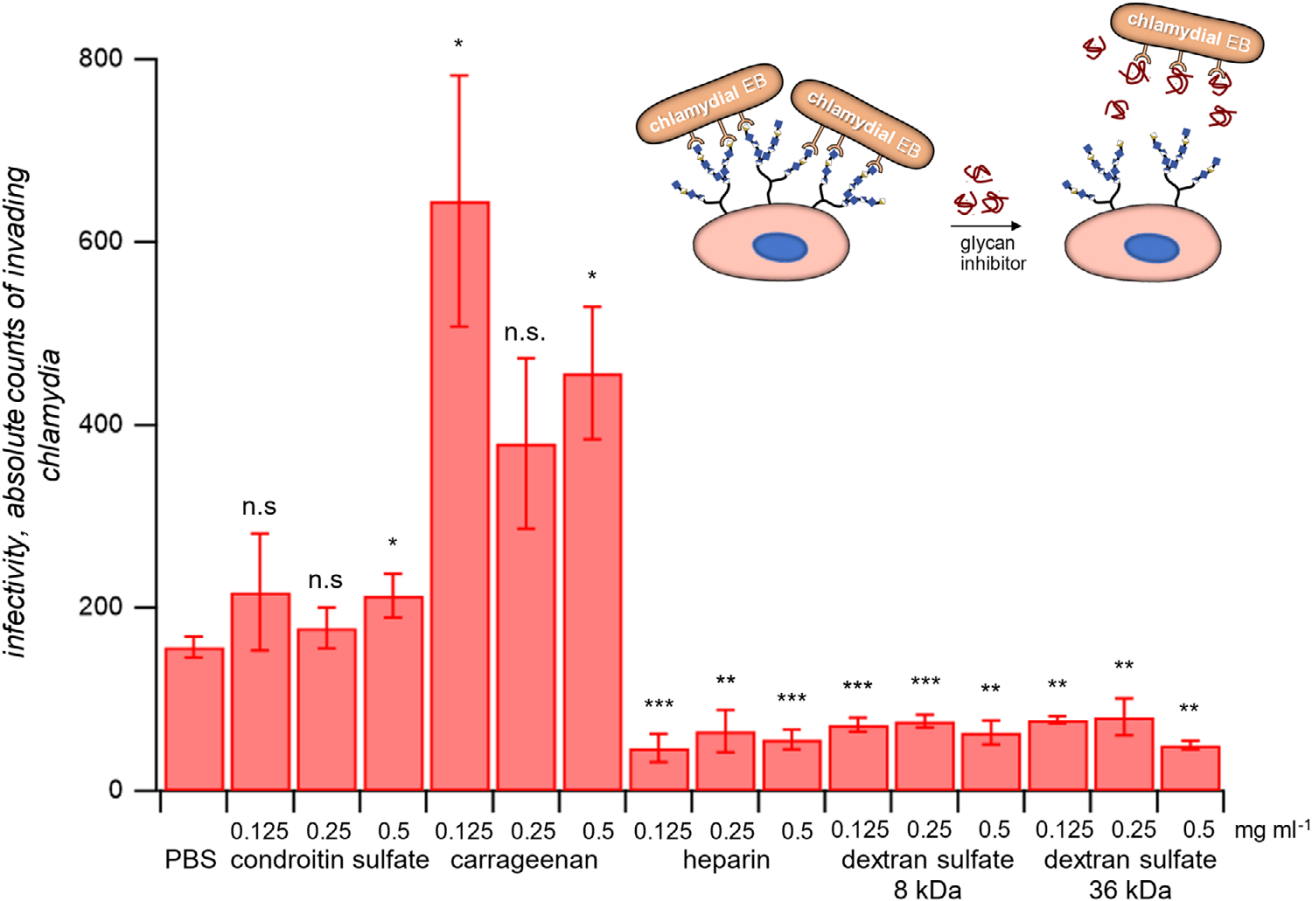
Attachment of chlamydial EBs to HEp‐2 cells as a measure of the *Cpn* infectivity in presence of various sulfated GAGs. Measurements for each condition were conducted three times. Data are presented as averages and the statistical significance was evaluated using t‐test (*<0.05, **<0.01, ***<0.001, ****<0.0001; ns: not significant).

Finally, we studied the effect of the GAG collection in the infection of HEp‐2 cells with infectious *Chlamydia pneumoniae* (*Cpn*) EBs (see introduction). The EBs were pre‐incubated with the various glycans and then added to a human cell layer. The cell attached‐EBs were visualized via immunostaining and counted in accordance to previous work (**Figure** **7**).^[^ ^12^ ^]^ In line with the results from the OmcB binding assays, carrageenan and chondroitin sulfate showed no inhibition of EB attachment to HEp‐2 cells and supports earlier studies.^[^ ^54^ ^]^ In contrast, inhibition of EB binding was strongest in case of heparin, reducing the number of attached EBs by up to 65% in comparison to samples without GAGs (PBS), again in line with earlier studies.^[^ ^54^ ^]^ The dextran sulfates showed a similar inhibitory effect, albeit the number of attached EB was somewhat higher compared to EBs treated with heparin. Overall, heparin demonstrated a more potent inhibitory effect with comparable sulfation levels and molecular weights, indicating that specific molecular interactions were at play.

Importantly, the inhibition of EB attachment was not complete, although the concentration of the heparin and dextran was well above the IC_50_, where no OmcB^GFP^ binding to HEp‐2 cell occurred. This hints at EB binding modes that do not require the presence of GAGs at the cell surfaces. For example, binding may take place at the entry receptors and via other adhesins. Another surprising finding was that the addition of carrageenan leads to an increase of EB attachment, although this high molecular weight GAG (200‐800 kDa) showed considerable inhibition of OmcB binding. In contrast to our finding, carrageenan was typically shown to act as an inhibitor of *Chlamydia* infections.^[^ ^55^, ^56^ ^]^ However, these studies investigated *C. trachomatis* instead of *Cpn* and also used different cell lines. An increase of EB‐cell attachment could be explained by interactions of the carrageenan with both EBs and HEp‐2 cells. Some studies suggest binding of carrageenan with human cells, but in other contexts and different cell types.^[^ ^57^, ^58^ ^]^ If such interactions are present, the high molecular weight GAG may bridge to EBs leading to their attachment. Such bridging interactions are present between colloidal particles (like EBs) and high molecular weight polyelectrolytes.^[^ ^59^ ^]^ This is a potentially relevant result in the context of bacterial infections, warranting a more detailed study in the future.

## 3 Conclusion

This study highlights the significant role of sulfated glycosaminoglycans (GAGs) as inhibitors in bacterial infections, emphasizing the importance of both molecular weight and sulfation patterns. The interaction between GAGs and the adhesin OmcB of *Chlamydia pneumoniae* serves as a primary focus, revealing key insights into the mechanics of bacterial pathogen adhesion and infection processes. Our findings demonstrate that increased sulfation and higher molecular weight enhance the binding affinity of GAGs to OmcB, which in turn reduces bacterial infectivity. Notably, heparin, despite its relatively lower molecular weight and sulfation degree, shows a remarkably high inhibitory effect on OmcB, suggesting that specific molecular architectures play a critical role in effective inhibition. The implications of these results extend beyond the specific interaction between Chlamydia and GAGs. The study sheds light on the broader context of pathogen adhesion mechanisms and offers potential avenues for the development of novel therapeutic strategies. By targeting the initial step of infection, GAG mimetics and other multivalent molecules present promising options in the fight against bacterial infections, particularly in the era of rising antibiotic resistance. Moreover, our research underscores the complexity of pathogen‐host interactions and the necessity for a nuanced understanding of the structural configurations of GAGs. While the potential of GAGs as inhibitors is clear, further investigations are warranted to optimize their specificity and efficiency while minimizing unwanted side effects, such as anticoagulant activity. In conclusion, this study not only advances our understanding of the biochemistry underlying bacterial infections but also points toward innovative approaches in infection prevention and treatment. The fine‐tuning of GAG structural properties opens up exciting possibilities for the development of new anti‐infection agents, marking a significant step forward in the field of microbial pathogenesis and host defense mechanisms.

## 4 Experimental Section

### OmcB^GFP^ Expression and Purification

Plasmid pDS91 (Figure S16, Supporting Information) encoding OmcB^GFP^ (= GFP‐OmcB‐BD) were transformed into the *E. coli* strain BL21 for expression using electroporation. One liter LB medium (containing 10 g bacto‐tryptone, 5 g yeast extract, 5 g NaCl) supplemented with 15 µg ml^−1^ kanamycin) was inoculated with transformed *E. coli* cells to an OD600 of 0.1 and incubated at 37 °C with shaking at 140 rpm until an OD600 of 0.7 to 1 was reached. Protein expression was then induced by adding isopropyl‐β‐D‐thiogalactopyranoside (IPTG) to a final concentration of 1 mM. After 4 hours, the cells were harvested by centrifugation at 5,000 rpm for 10 minutes (Avanti J‐20, Beckman Coulter).

For protein purification under native conditions, the cell pellet from a 1 litre *E. coli* culture was resuspended in 20 ml of lysis buffer (0.2 mg ml^−1^ lysozyme, 1 mM PMSF, 0.3% sodium lauroyl sarcosinate, 0.3% Triton X100, 1x Roche protease inhibitor cocktail, in PBS) and incubated overnight at 4 °C on a rotating wheel. The next day, the suspension was sonicated three times on ice for 10 seconds each, followed by centrifugation at 24,000 rpm for 20 minutes at 4 °C (Avanti J‐20, Beckman Coulter). The supernatant was then filtered and the protein purified using a using a Ni‐NTA HpP Column (HisTrapTM HP) on an ÄKTA chromatography system (Fast Protein Liquid Chromatography). Washing steps involved PBS with 20 mM and 40 mM imidazole, and the protein was eluted with 500 mM imidazole in PBS. The protein was eluted with a concentration of 500 mM of imidazole. The protein concentration was determined using the Bradford assay, and purity was assessed by Coomassie‐stained SDS‐PAGE and Western blot analysis. The protein solution was transferred to a dialysis tube (VISKING, SERVA) and dialyzed against PBS at 4 °C. Finally, the protein solution was flash‐frozen in liquid nitrogen and stored at −80 °C. PBS (phosphate buffered saline): 137 mM NaCl, 2.7 mM KCl, 10 mM Na2HPO4, 1.8 mM KH2PO4 in ddH2O.

### Western Blot Analysis

The proteins separated by SDS‐PAGE were transferred to a polyvinylidenfluorid (PDVF) membrane. For this purpose, the PVDF membrane was activated in MeOH for 10 min at RT. The membrane and Whatman paper sheets were then equilibrated in transfer buffer (25 mM Tris, 150 mM Glycine, 20% Methanol, 0.05% SDS in H_2_O). Protein transfer was carried out using a Pierce G2 Fast Blotter at 25 V, 1A for 20 – 30 min as recommended by the manufacturer. The PVDF membrane was treated for 30 min at RT at 40 rpm in blocking solution (5% BSA, 0.05% Tween 20, in PBS pH 7.4).

For protein immune detection, the PVDF membrane was incubated for 1 h at RT with the primary antibody diluted in blocking solution; mouse anti‐GFP antibody (MA5‐15256) from Thermo Scientific (1:2000 dilution), mouse anti‐β‐actin antibody from Sigma‐Aldrich (1:1000 dilution). Next, the PVDF membrane was washed three times in PBS at 40 rpm for 10 min each, followed by incubation in blocking solution for 1 h at RT with the secondary anti‐mouse antibody coupled to alkaline phosphatase (#A3562, 1:30 000) from Sigma‐Aldrich. The PVDF membrane was washed three times in PBS as 40 rpm for 10 min each and then incubated in the dark in 20 ml detection buffer (0.1 M Tris/HCl pH9,5, 0.1 M NaCl, 50 mM MgCl2, in H_2_O) with 33 µl BCIP solution (5% (w/v) 5‐bromo‐4‐chloro‐3‐indolyl‐phosphate (BCIP) in Dimethylformamide) and 33 µl NBT solution (5% (w/v) Nitro blue tetrazolium (NBT) in 70% DMF solution) each until bands appeared.

### GAG Synthesis and Characterization

The synthesis of the polymannose samples were shown in the supporting information (see images S1–S9, Supporting Information). Briefly, the synthesis started by preparing a mannose monomer with a N‐hydroxyethylacrylamide group for polymerization.^[^ ^60^ ^]^ The photopolymerization was conducted under UV‐light with TPO (Diphenyl(2,4,6‐trimethylbenzoyl)phosphinoxid)as an initiator in DMF. Sulfation was conducted according to previously developed protocols.^[^ ^25^ ^]^ The sulfation degree of all GAGs was determined via atom absorption spectroscopy using a Vario Micro Cube (Elementar Analysensysteme GmbH, Langenselbold, Germany).

### Microplate Surface Preparation Polyelectrolyte Multilayers

Greiner Bio‐One 96‐well microtiter plates (Sensoplate Microplates, PS, F‐Bottom, Glass Bottom) were used. The plates were cleaned using UV‐ozone for 30 minutes. A solution with a concentration of 1 mg ml^−1^ Polyethyleneimine (Mw 750,000 g mol^−1^, branched, Sigma Aldrich) in water was pipetted into the wells and incubated for 1 h at room temperature. The wells were rinsed by immersing the microplate in ultrapure water for 20 seconds twice. Afterwards, 1 mg ml^−1^ heparin in 0.5 M NaCl was pipetted into the wells and incubated for 1 hour at room temperature. The wells were then cleaned twice by immersion in ultrapure water.

### Microplate Reader Assay

The CLARIOstar microplate reader from BMG Labtech was used for the fluorescence measurements of the inhibition curves (Greiner Bio‐One 96‐well microtiter plates were used for the experiments). Phosphate buffered saline (PBS) was prepared with a final concentration of 0.01 M phosphate buffer, 0.0027 M potassium chloride and 0.137 M sodium chloride, pH 7.4 at 25 °C.

A solution of 0.2 mg ml^−1^ OmcB^GFP^ in PBS containing 0.02 vol% Tween20 and a dilution series of the GAGs to be tested in PBS containing 0.02 vol% Tween20 were prepared. The OmcB and the GAG solutions were combined at a ratio of 1:1 and incubated for 10 min and then pipetted into the wells in triplicates. The microwell plate was incubated for 1 h at room temperature. Next, the non‐bound OmcB^GFP^ in the supernatant was removed from the wells by pipetting and carefully washing three times with PBS buffer (3 × 200 µl). The wells were filled with 120 µl PBS buffer and the fluorescence signal was measured with the microplate reader.

### OmcB Binding Studies

Confluent layers of HEp‐2 cells in 24‐well plates were washed with cold Hanks’ balanced salt solution (HBSS). Subsequently, 300 µl of a 200 µg ml^−1^ solution of GFP‐ OmcB^GFP^ was prepared in cell culture medium. OmcB^GFP^ was pre‐incubated for 1 h with the different GAGs at 250 µg ml^−1^ or 500 µg ml^−1^. The total protein concentration was 200 µg ml^−1^. The cells were exposed to the OmcB^GFP^ solution for 60 min at 4 °C, and then washed three times with HBSS. Fluorescence microscopy was conducted using the GPF channel and DAPI staining to visualize OmcB^GFP^ binding. Next, the cells were detached by incubation with 200 µl of Cell Dissociation Solution Non‐enzymatic 1 x (Sigma‐Aldrich 10 min, 37 °C), transferred to a reaction tube, and pelleted by centrifugation for 5 min at 1000 × g (Heraeus Biofuge Primo R). The cell dissociation solution was then removed and the pellet was resuspended in 97.5 µl PBS, 37.5 µl protein blue marker and 15 µl DTT. After incubation for 10 min at 100 °C, all samples were analyzed via Western blots.

### Growth of Chlamydia Pneumoniae

Cultivation of adherent human HEp‐2 cells, CHO‐WT or CHO‐pgsA or CHO‐pgsD cells was carried out in 25 cm^2^ cell culture flasks at 37 °C and 6% CO_2_ in DMEM+5 cell culture medium (500 ml DMEM GlutaMAX (Gibco), 50 ml FCS heat inactivated at 56 °C for 1 h (Gibco), 5 ml MEM non‐essential amino acids (100×) (Gibco), 5 ml MEM vitamins (100×) (Invitrogen), 5 mlAmphotericin B (250 µg ml^−1^) (Life technologies), 500 µl Gentamycin (50 mg ml^−1^) (Invitrogen)).

*Chlamydia pneumoniae* (*Cpn*) was an obligate intracellular pathogen and relies on host cells for its infection cycle. Therefore, monolayers of HEp‐2 cells were used for cultivating *Cpn* in cell culture medium DMEM+5 which was supplemented with 12 µl ml^−1^ Cycloheximide, inhibiting HEp‐2 replication (termed Chlamydia culture medium).

HEp‐2 cells were grown in cell culture medium to confluence in 25 cm^2^ flasks. A *Cpn* cell suspension (≈10^7^ inclusion forming units (IFU)/ml) in SPG buffer (75 g Sucrose, 0.52 g KH_2_PO_4_, 1.53 g Na_2_HPO_4_, 0.72 g Glutamic acid, in 1 liter H_2_O, pH 7.5) was added to each flask and flasks were centrifuged at 1560 x g and 30 °C for 60 min (Rotana 460R). Cells were incubated for 1 h at 37 °C and 6% CO_2_. Cell culture medium was replaced with 5 ml of fresh Chlamydia culture medium. Infection proceeded at 37 °C and 6% CO_2_ for 3 days. HEp‐2 cells were detached form the flask using a sterile cell scraper and the cell suspension was collected in a 50 ml centrifuge tube and sonicated for 45 sec with 40% power (Ultrasonic Homogenizer Sonopuls HD2200, Bandelin) to disrupt cells and centrifuged for 10 min at 2670 rpm (Rotana 460R). Supernatant was collected in a new tube and centrifuged again for 10 min at 2670 x g (Rotana 460R). This supernatant was either used for a new round of infection or frozen in SPG (dilution 1:1) at −80 °C.

### Infection Assay

A confluent cell layer (1 × 10^6^ cells per well on a coverslip, CHO‐WT or CHO‐pgsA or CHO‐pgsD cells) was washed once with Hanks buffered salt solution (HBSS *Cpn* cells at a multiplicity of infection (MOI) of 20 were mixed with a specific glycan (500 µg ml^−1^, 250 µg ml^−1^ or 125 µg ml^−1^) in cell culture medium and incubated for 1 hour at 4 °C. After incubation, the mixture was applied to the cells and incubated for 2 hours at 37 °C with 6% CO_2_. Subsequently, the solution was replaced with 1 ml of Chlamydia culture medium. After 48 hours at 37 °C in 6% CO_2_, cells were fixed with 96% methanol for 10 minutes at RT, washed with PBS, and either stored at 4 °C or stained for direct immunofluorescence using 30 µl of a fluorescein‐conjugated monoclonal Chlamydia‐specific anti‐lipopolysaccharide antibody (Pathfinder; Bio‐Rad) solution (1:6 diluted in PBS). The inclusion bodies within the HEp‐2 cells harboring the chlamydial cells were quantified by counting 10 microscopy fields of each view. The result was reported as a percentage relative to the PBS‐treated negative control.

## Conflict of Interest

The authors declare no conflict of interest.

## Supporting information



Supporting Information

## Data Availability

The data that support the findings of this study are available in the supplementary material of this article.
