# Supplementary material for: Sulfated Glycosaminoglycans as Inhibitors for Chlamydia Infections: Molecular Weight and Sulfation Dependence
Source: Macromol Biosci. 2025 Jan 21;25(4):2400443. doi: 10.1002/mabi.202400443 (PMC11995835; doi:10.1002/mabi.202400443)
Supplement: Supplementary file 1 — Supporting Information [file MABI-25-2400443-s001.pdf]

Macromolecular Bioscience

Supporting Information

for *Macromol. Biosci.*, DOI 10.1002/mabi.202400443

Sulfated Glycosaminoglycans as Inhibitors for Chlamydia Infections: Molecular Weight and Sulfation Dependence

Sebastian Wintgens, Janita Müller, Felicitas Drees, Dominik Spona, Lorand Bonda, Laura Hartmann, Johannes H. Hegemann and Stephan Schmidt**

Supporting information

Sulfated glycosaminoglycans as inhibitors for chlamydia infections: molecular weight and sulfation dependence

Sebastian Wintgens, Janita Müller, Felicitas Drees, Dominik Spona, Lorand Bonda, Laura Hartmann, Johannes H. Hegemann and Stephan Schmidt

Table of contents

Elemental Analysis 1
pMan-monomer synthesis 2
Polymer synthesis pMan8.3 4
Polymer synthesis pMan19 6
Polymer synthesis pMan83 7
SEC analysis 9
pMan sulfation to give pManSulf20, pManSulf42, pManSulf198 9
Layer-by-layer buildup of SiO_2-polyethyleneimine-heparin surfaces and direct $OmcB^{GFP}$ binding measurements via quartz crystal microbalance (QCM) 10
$OmcB^{GFP}$ binding inhibition assay by fluorescence microscopy readout 10
Supplementary data on direct OmcB binding to different cell lines 11
Plasmid pDS91 14

Elemental Analysis

The proportions of carbon, hydrogen, nitrogen, and sulfur were measured with a Vario Micro Cube from Elementar Analysensysteme GmbH GmbH. These measurements were conducted by the Institute for Pharmaceutical and Medicinal Chemistry at Heinrich-Heine University Düsseldorf.

Table S1 Ratio of carbon, hydrogen, nitrogen, and sulfur as measured by elemental analysis

	Heparan sulfate	Heparin 1-5 kDa	Heparin 4-6 kDa	Heparin 17-19 kDa	Dextran Sulfate 8 kDa	Dextran Sulfate 36-50 kDa	Chond roitin Sulfate	Carrage enan	pManSulf 20 kDa	pManSulf 42 kDa	pManSulf 198 kDa
%C	30.86	21.5	21.0	20.5	14.3	14.2	28.8	24.5	18.5	17.2	17.7
%H	4.79	3.7	3.6	3.5	2.8	2.7	4.7	4.0	3.3	3.3	3.3
%N	2.89	2.0	1.9	1.8	0	0	2.5	0	2.3	1.7	1.7
%S	6.31	10.7	9.8	8.9	14.6	16.3	4.8	10.7	16.6	15.5	16.2

pMan-monomer synthesis

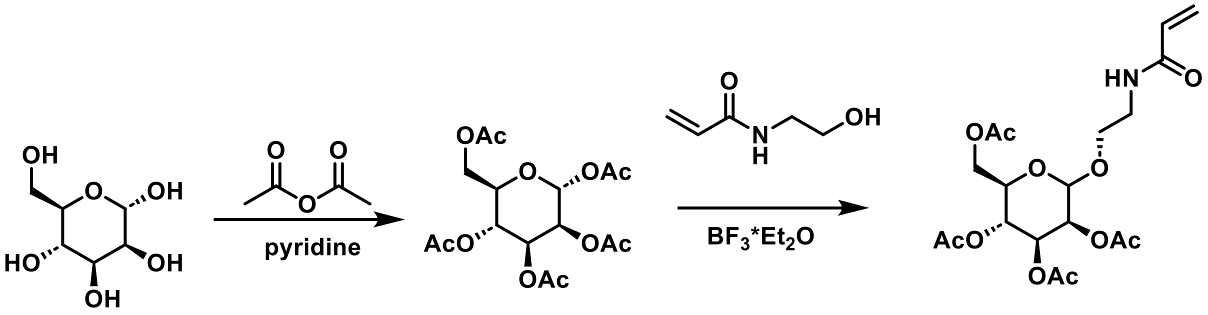

The synthesis of the mannose monomer was adapted by Wilkins et al.[60] The acetylated mannoseacrylamide monomer was synthesized by dissolving D-mannose in a mixture of a 1:1 (v/v) mixture of pyridine/acetic anhydride [20 mL/g] and stirring at room temperature overnight. After diluting with ethylacetate the mixture was extracted three times with 1M HCl solution. Evaporation of ethylacetate resulted in 1,2,3,4,6-penta-O-acetyl-α-D-mannopyranose. Pentaacetylated mannose (1.0 eq.) and N-hydroxyethylacrylamide (1.2 eq.) were dissolved in DCM [2 mL/mmol] and flushed with argon gas for 10 minutes. BF3*Et2O (10.0 eq.) was added through a syringe and the mixture stirred at room temperature overnight. The reaction solution was washed three times with brine and the organic phase dried with MgSO4. The solvent was removed, which resulted in pure acetylated mannosemonomer (AcO-ManAAm) with a relative purity of 98 % and a yield of 78 %.

ESI-MS: m/z calculated for $C_{19}H_{27}NO_{11}$ $[M+H]^+$ 446.16 and $[M+Na]^+$ 468.15; found $[M+H]^+$ 446.46 and $[M+H]^+$ 468

Analytics

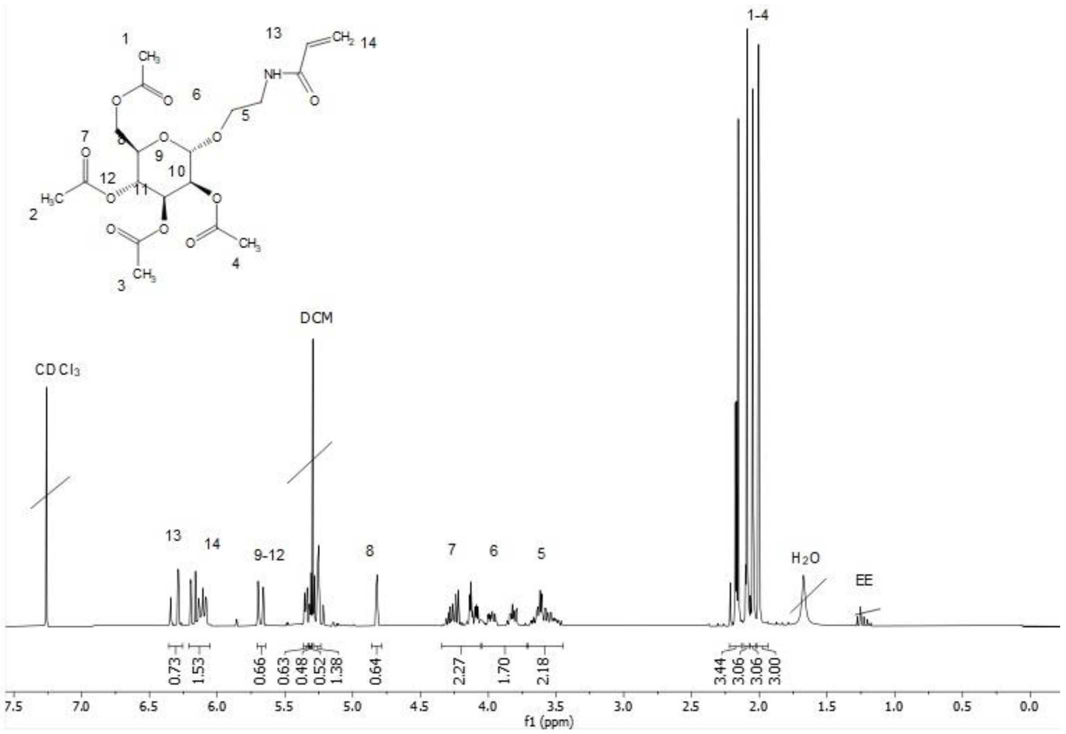

Figure S1 ^{1}H NMR spectrum (600 MHz, CD_3OD) of pMan-monomer.

^{1}H-NMR (300 MHz, $CDCl_3$): δ (ppm) 2.00-2.16 (s, 12H, C**H**$_3$ H1-4), 3.46-3.61 (m, 2H, C**H**$_2$ H5), 3.79-4.02 (m, 2H, C**H**$_2$, H6), 4.06-4.23 (m, 2H, C**H**$_2$, H7), 4.82 (s, 1H, C**H**, H8), 5.22-5.69 (m, 4H, C**H**, H9-12), 6.15 (dd, ^{2}J=10.2 Hz, ^{3}J=17.1 Hz, 2H, C**H**$_2$, H14), 6.32 (dd, ^{2}J=1.2 Hz, ^{3}J=17.1 Hz, 1H C**H**, H13)

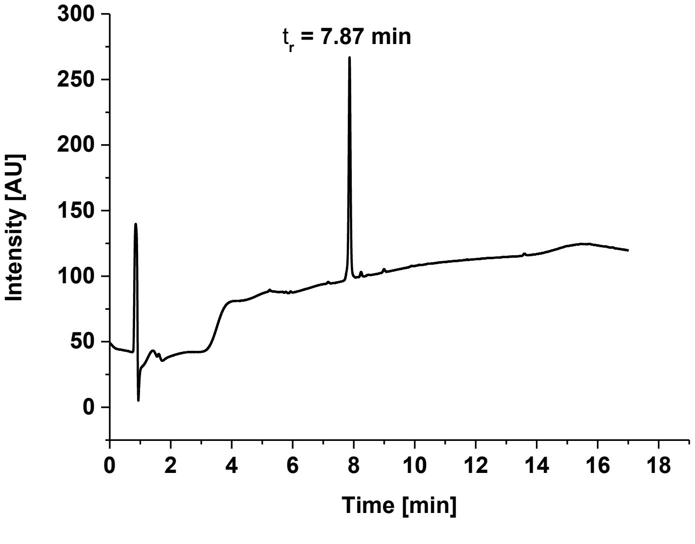

Figure S2 RP-HPLC of pMan-monomer (A: 95% H_2O/ 5% MeCN/ 0.1% Formic Acid; 100% A -> 50% A in 30 min): t_r = 7.87 min.

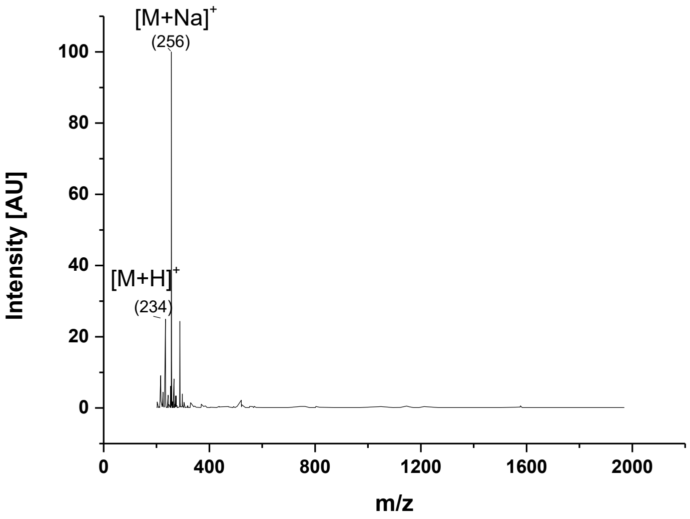

Figure S3 ESI-MS of pMan-monomer.

Polymer synthesis pMan8.3

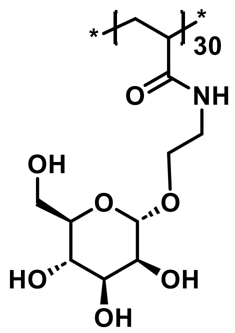

222.7 mg of pMan-monomer (0.5 mmol) and TPO (3 mol%, 0.015 mmol) were dissolved in DMF [10 wt.%] and the solution was flushed with Argon for 10 minutes and irradiated with UV-light (405 nm wavelength, with an intensity 45.2 mW/cm^2). After an hour, the irradiation was stopped and 5 mL NaOMe (0.2 M) in MeOH was added to the polymer solution and stirred one hour at room temperature. Solid matter that has already precipitated and the residual solution was precipitated in diethyl ether. The precipitated polymer was dissolved in H_2O, dialyzed against distilled water (three cycles, 2 kDa) and subsequently lyophilized.

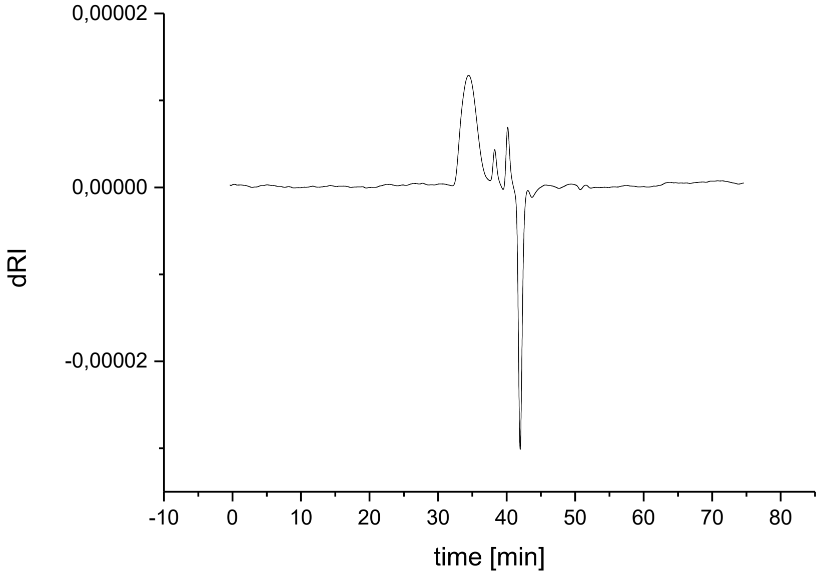

Figure S4 Size exclusion chromatography (SEC) elution profile of pMan17 in H_2O

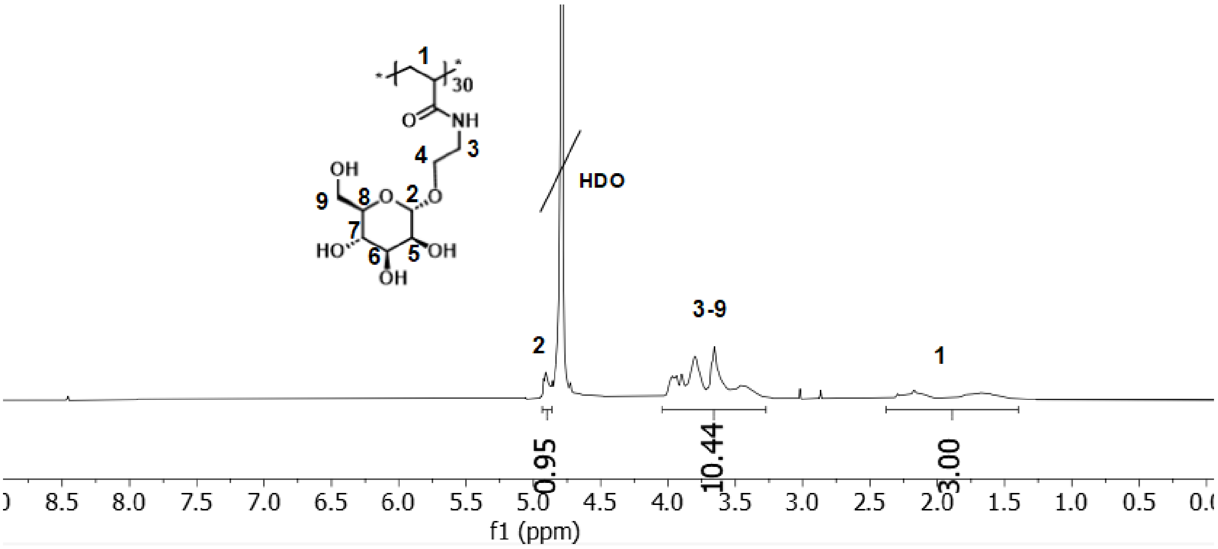

Figure S5 NMR spectrum (600 MHz, D_2O) of pMan17.

^{1}H-NMR (600 MHz, D_2O) δ [ppm] 4.93-4.88 (m, **2,** D_2O overlapping), 4.04-3.25 (m, **3-9**), 2.37-1.38 (m, **1**).

Polymer synthesis pMan19

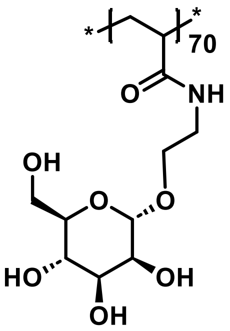

222.7 mg of monomer pMan-monomer (0.5 mmol) and TPO (1.4 mol%, 0.007 mmol) were dissolved in DMF [10 wt.%] and the solution was flushed with Argon for 10 minutes and irradiated with UV-light (405 nm wavelength, with an intensity 45.2 mW/cm^2). After an hour, the irradiation was stopped and 5 mL NaOMe (0.2 M) in MeOH was added to the polymer solution and stirred one hour at room temperature. Solid matter that has already precipitated and the residual solution was precipitated in diethyl ether. The precipitated polymer was dissolved in H_2O, dialyzed against distilled water (three cycles, 2 kDa) and subsequently lyophilized.

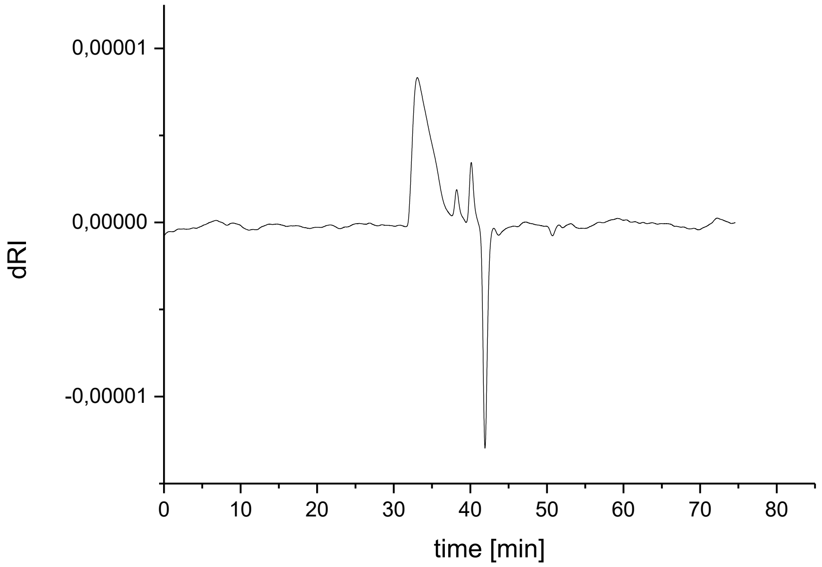

Figure S6 Size exclusion chromatography (SEC) elution profile of pMan44 in H_2O

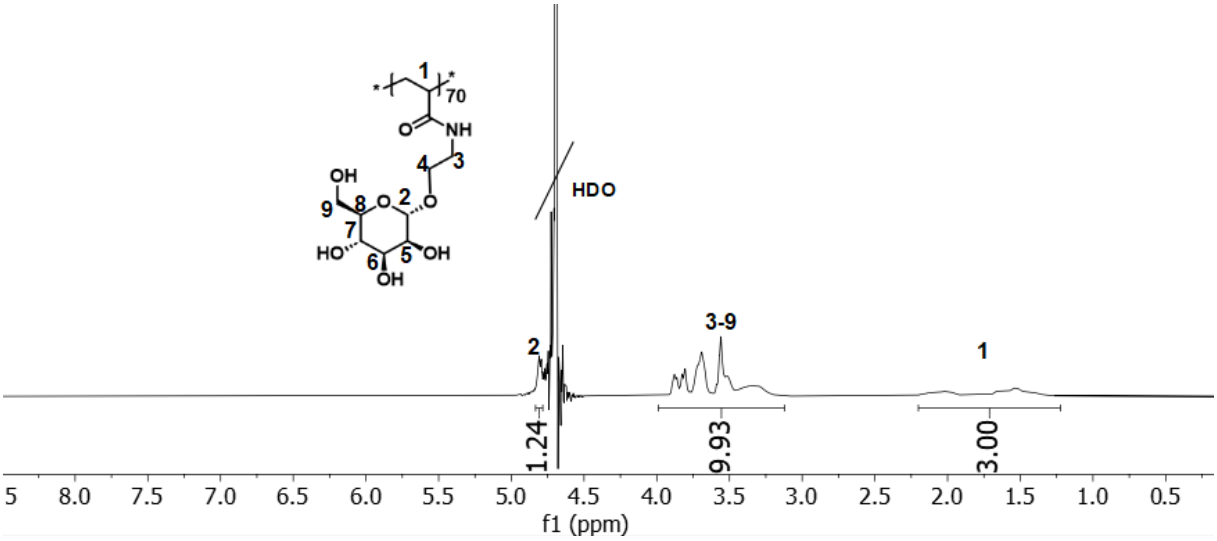

Figure S 7 ^{1}H NMR spectrum (600 MHz, D_2O) of pMan44.

^{1}H-NMR (600 MHz, D_2O) δ [ppm] 4.85-4.77 (m, **2,** D_2O overlapping), 3.98-3.11 (m, **3-9**), 2.19-1.22 (m, **1**).

Polymer synthesis pMan83

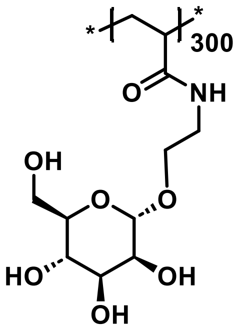

445.4 mg of monomer pMan-monomer (1 mmol) and TPO (0.33 mol%, 0.0033 mmol) were dissolved in DMF [10 wt.%] and the solution was flushed with Argon for 10 minutes and irradiated with UV-light (405 nm wavelength, with an intensity 45.2 mW/cm^2). After an hour, the irradiation was stopped and 5 mL NaOMe (0.2 M) in MeOH was added to the polymer solution and stirred one hour at room temperature. Solid matter that has already precipitated and the residual solution was precipitated in diethyl ether. The precipitated polymer was dissolved in H_2O, dialyzed against distilled water (three cycles, 2 kDa) and subsequently lyophilized.

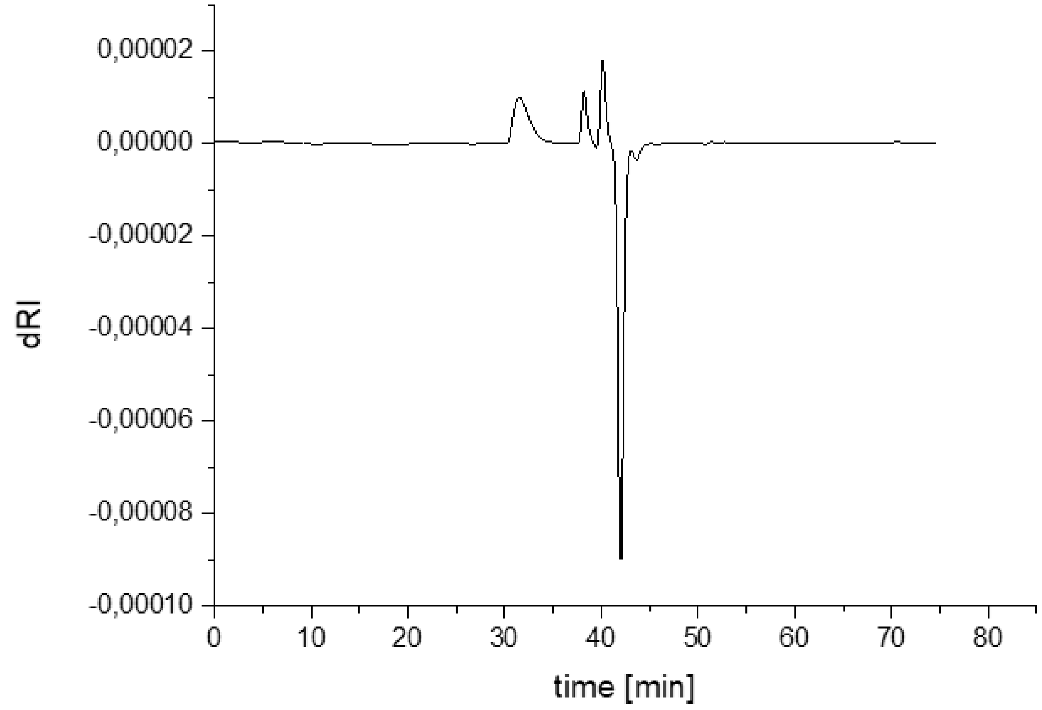

Figure S8 Size exclusion chromatography (SEC) elution profile of pMan190 in H_2O

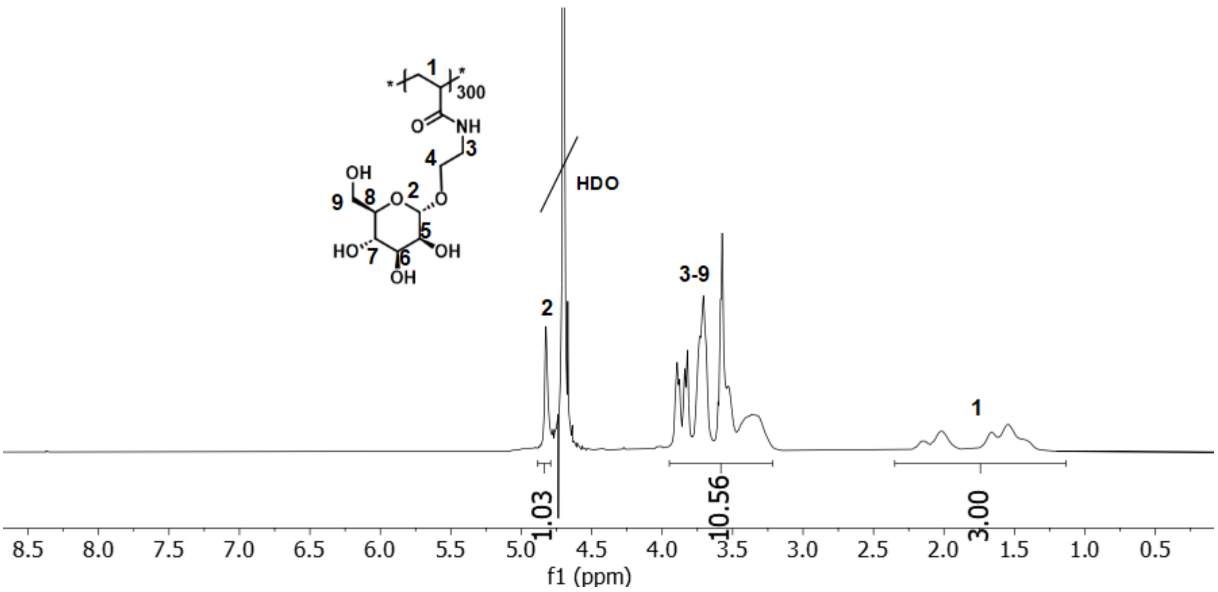

Figure S9 ^{1}H NMR spectrum (600 MHz, D_2O) of pMan190.

^{1}H-NMR (600 MHz, D_2O) δ [ppm] 4.87-4.79 (m, **2,** D_2O overlapping), 3.95-3.2 (m, **3-9**), 2.3-1.19 (m, **1**).

SEC analysis

SEC analysis was conducted with an Agilent 1200 series HPLC system and three aqueous SEC columns provided by Polymer Standards Service (PSS). The columns were two Suprema Lux analytical columns (8 mm diameter and 5 µm particle size) and one precolumn (50 mm, 2 x 160 Å of 300 mm and 1000 Å of 300 mm). The eluent was water.

pMan sulfation to give pManSulf20, pManSulf42, pManSulf198

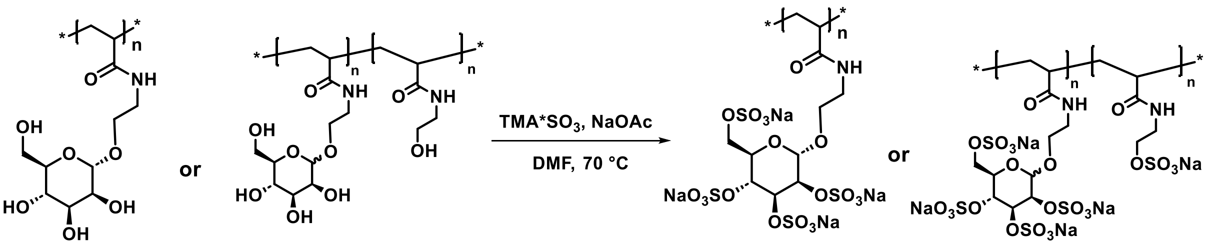

Sulfation of glycopolymers and glycol copolymers was performed as in an earlier published protocol.[25] TMA*SO3 (40 eq. per OH-group) was used as a sulfating agent and dissolved with the polymer in DMF and stirred for 18h at 70°C. After the solution was cooled down to room temperature, 20 eq. of aqueous sodium acetate solution (20 %) was added for quenching at 0°C. The solvent mixture was evaporated under reduced pressure, dialyzed (MWCO 5-10 kDa) and lyophilized.

The degree of sulfation was determined via elemental analysis. Theoretical values were calculated for 100% sulfation (without considering the end groups) and the degree of sulfation was calculated from the obtained values. For this purpose, the S/C ratio was calculated for optimal (100%) sulfation and also the S/C ratio was calculated for actual sulfatization. $((S/C)_{actual}/(S/C)_{optimal})*100$ forms the actual degree of sulfation.

After sulfation the samples have an increased molecular weight, thus the sample names become: pMan8.3 → pManSulf20, pMan19 → pManSulf42, pMan83 → pManSulf198 as given in the main text.

Layer-by-layer buildup of SiO2-polyethyleneimine-heparin surfaces and direct OmcB^{GFP} binding measurements via quartz crystal microbalance (QCM)

QCM measurements on pure silica chips to test the layer-by-layer buildup (PEI-heparin-OmcB) on a surface similar to the glass buttoned microwell plates. Measurements were conducted using a QCM-D instrument qCell T Q2 (3T analytic GmbH, Neuhausen ob Eck, Germany) with dual sensor channels and quartz chips from the same manufacturer. Prior to use, the chips were treated with air plasma for 30 seconds. All solutions were filtered through a 0.1 µm pore size filter, and degassed for 20 minutes via ultrasound. The polymers were dissolved in this solution at a concentration of 1 mg mL^{-1}. Before injecting the samples, the chips were stabilized with the different solutions, water or PBS, without polymer.

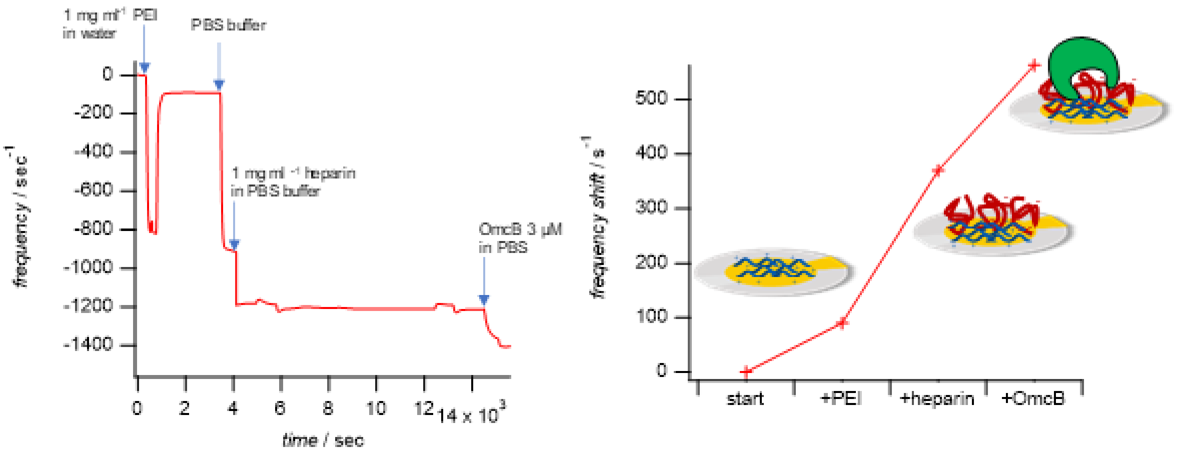

Figure S10 Left: QCM trace depicting the frequency changes upon addition of the different components for layer buildup. Right: calculated frequency shifts after the adsorption of PEI, heparin and OmcB, omitting the shifts for changing the buffer system.

OmcB^{GFP} binding inhibition assay by fluorescence microscopy readout

Heparin surfaces on glass surfaces (chambered coverslips, ibidi GmbH, Germany) were prepared as described in the experimental section. Fluorescence microscopy was conducted on an Olympus IX 73 equipped with an 60x oil objective and a Thorlabs M470L4 led for GFP excitation.

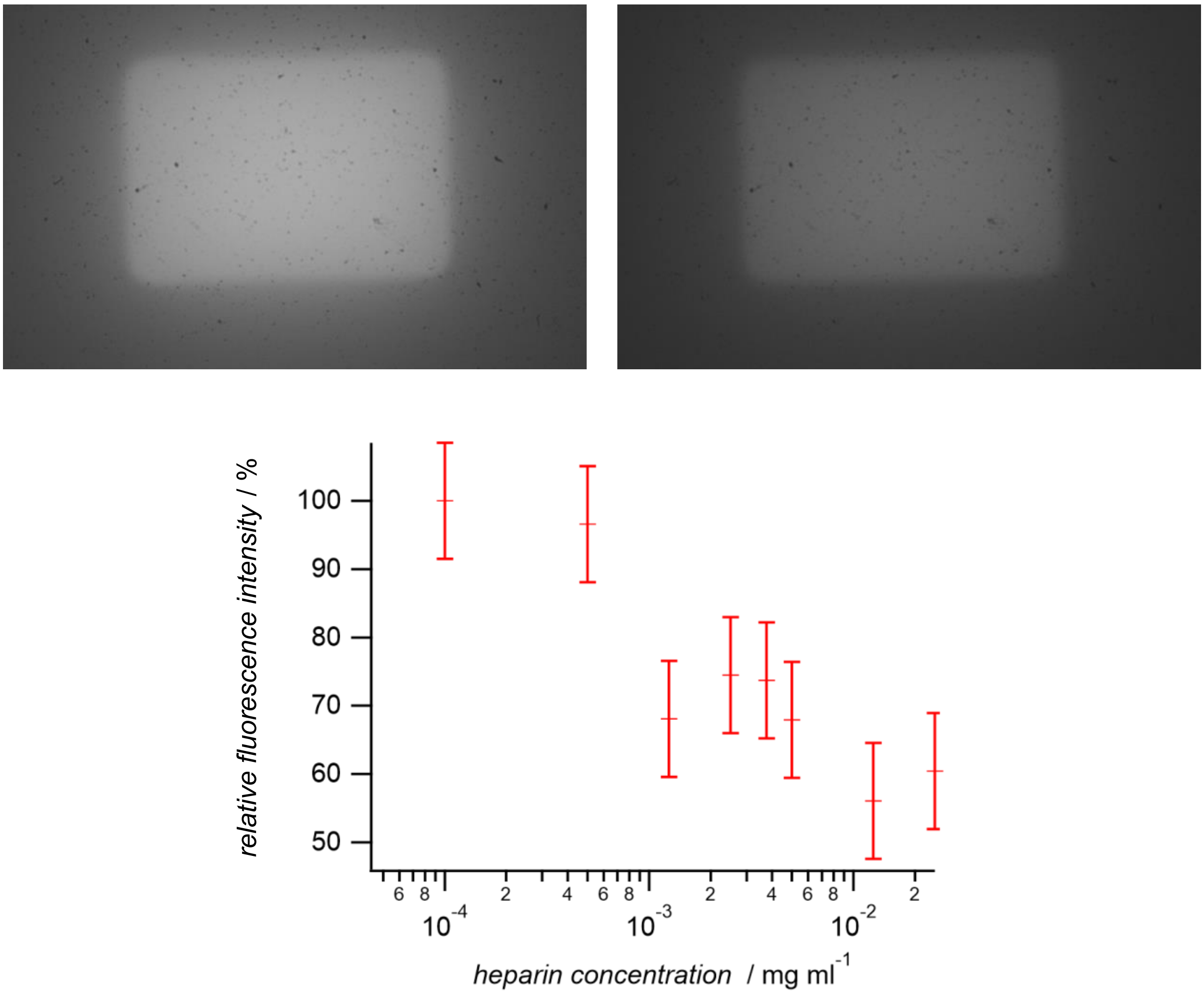

Figure S11 $OmcB^{GFP}$ binding on glass-polyethyleneimine-heparin surfaces, readout via fluorescence microscopy. Top: typical fluorescence microcopy image of the $OmcB^{GFP}$ without added heparin (left) and with 1 mg ml^{-1} heparin (right). Bottom: Averaged fluorescence intensities calculated from the grey values in the center of the image showing decreasing binding of OmcB to heparin surfaces upon adding heparin.

Supplementary data on direct OmcB binding to different cell lines

The cell lines HEp-2, CHO-WT, CHO-pgsA, and CHO-pgsD, were used in the OmcB binding studies. In contrast to the CHO-WT cell line, the CHO-pgsA and CHO-pgsD mutant cell lines were are deficient in HS synthesis), as specified in the main text. The cells were exposed to the $OmcB^{GFP}$ (200 µg ml^{-1}) solution for 60 min at 4 °C, either with or without added heparin (0.5 mg ml-1), and then washed three times with HBSS. For western blot analysis the cells were incubated in the same fashion for 15 min, 30 min, or 60. After incubation, the cell samples were lysed and probed for $OmcB^{GFP}$ and anti-actin for loading control.

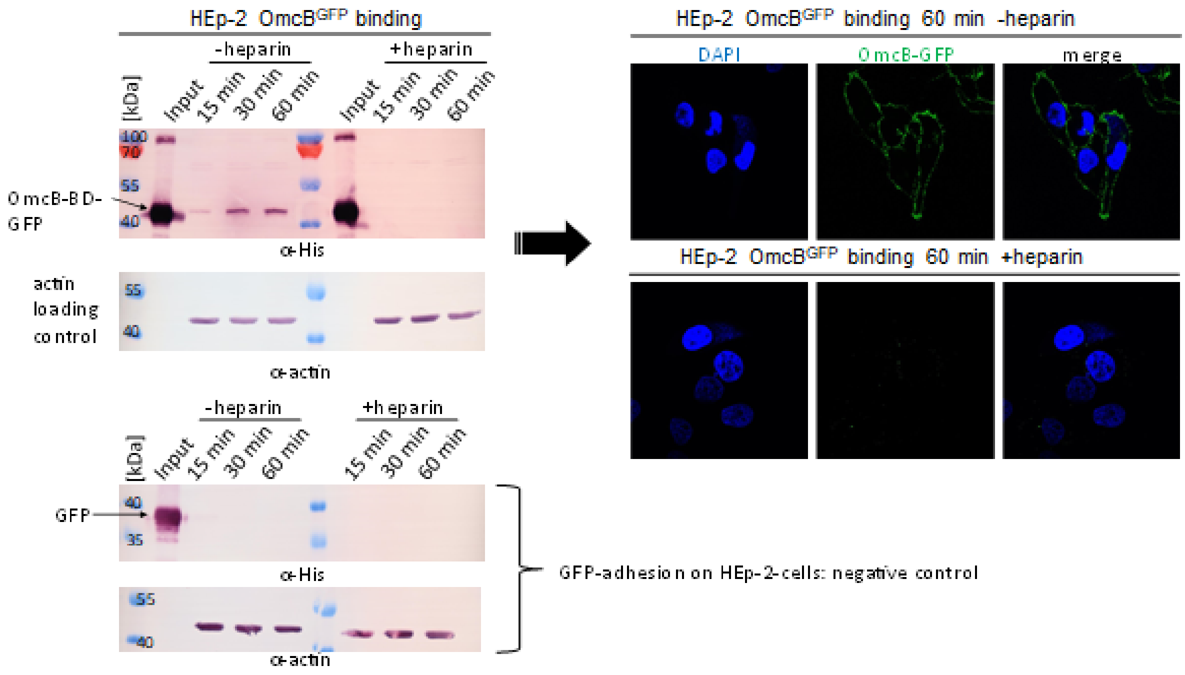

Figure S12 OmcB binding assays on Hep-2 cells

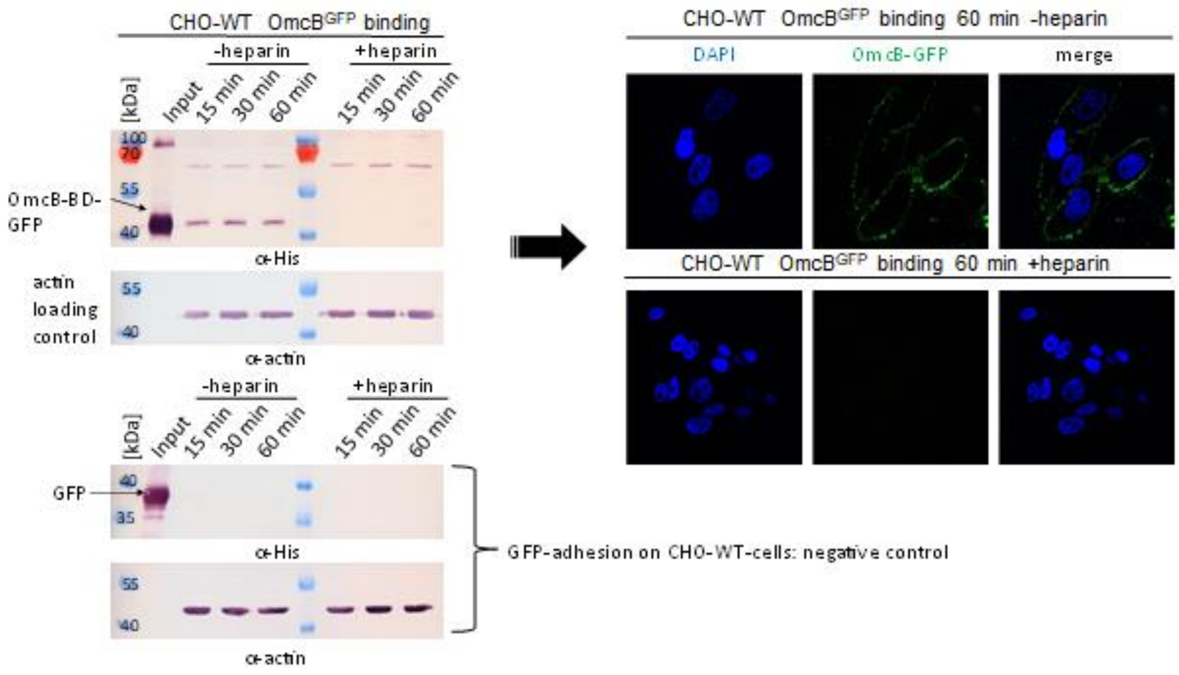

Figure S13 OmcB binding assays on CHO-WT cells

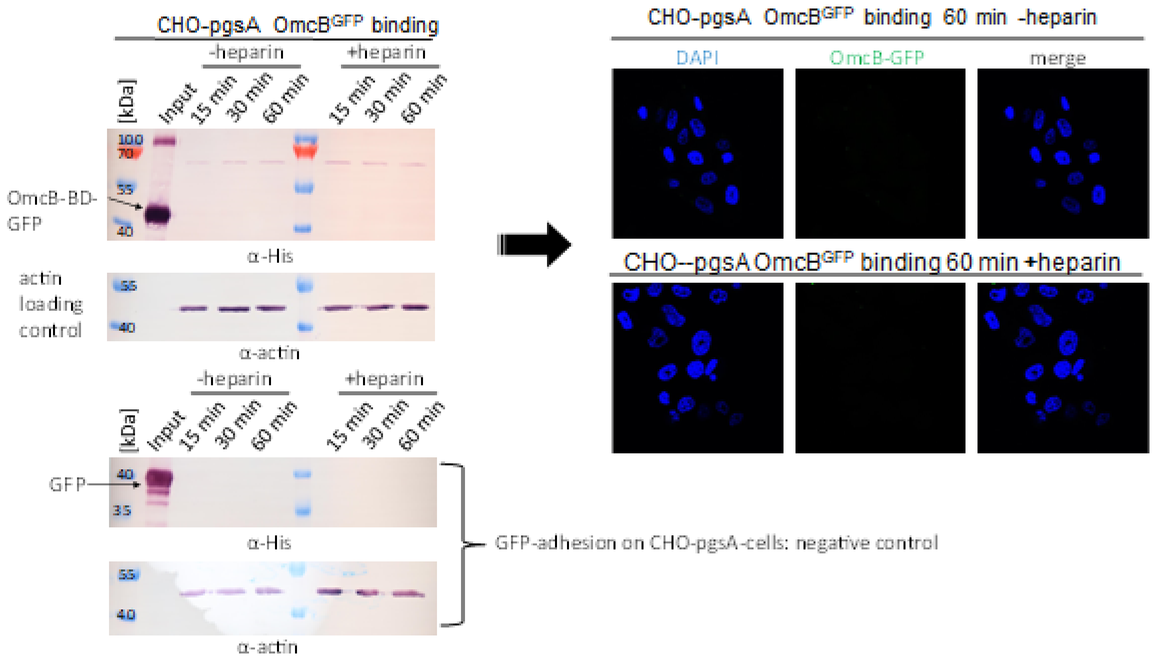

Figure S14 OmcB binding assays on CH-pgsA cells

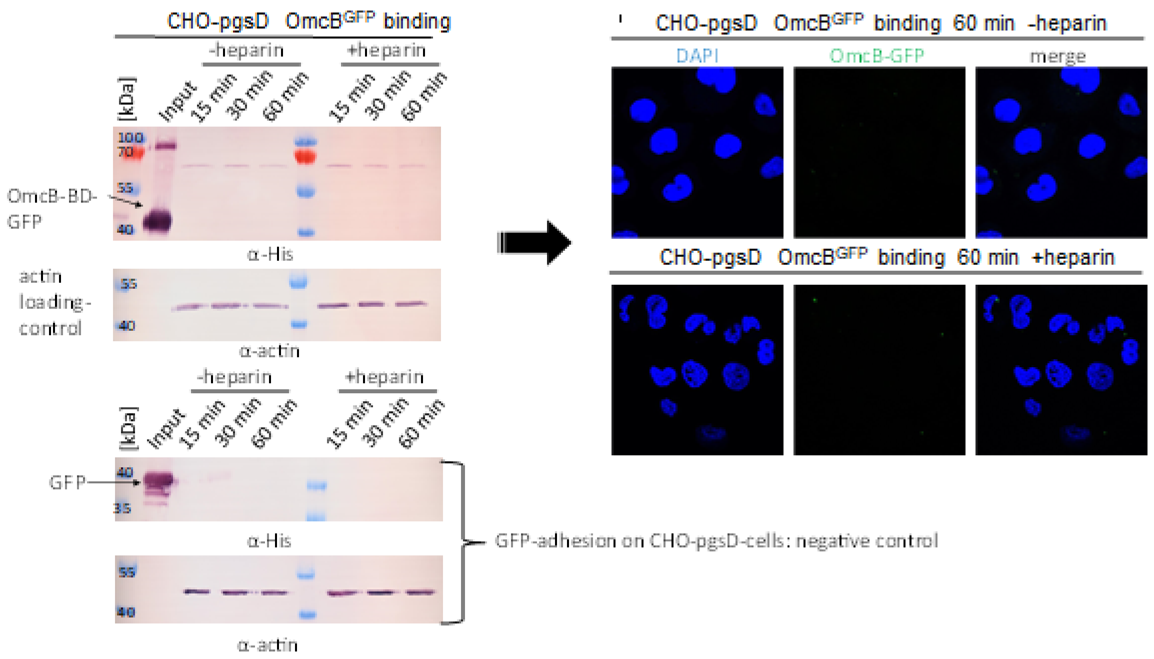

Figure S15 OmcB binding assays on CH-pgsD cells

Plasmid pDS91

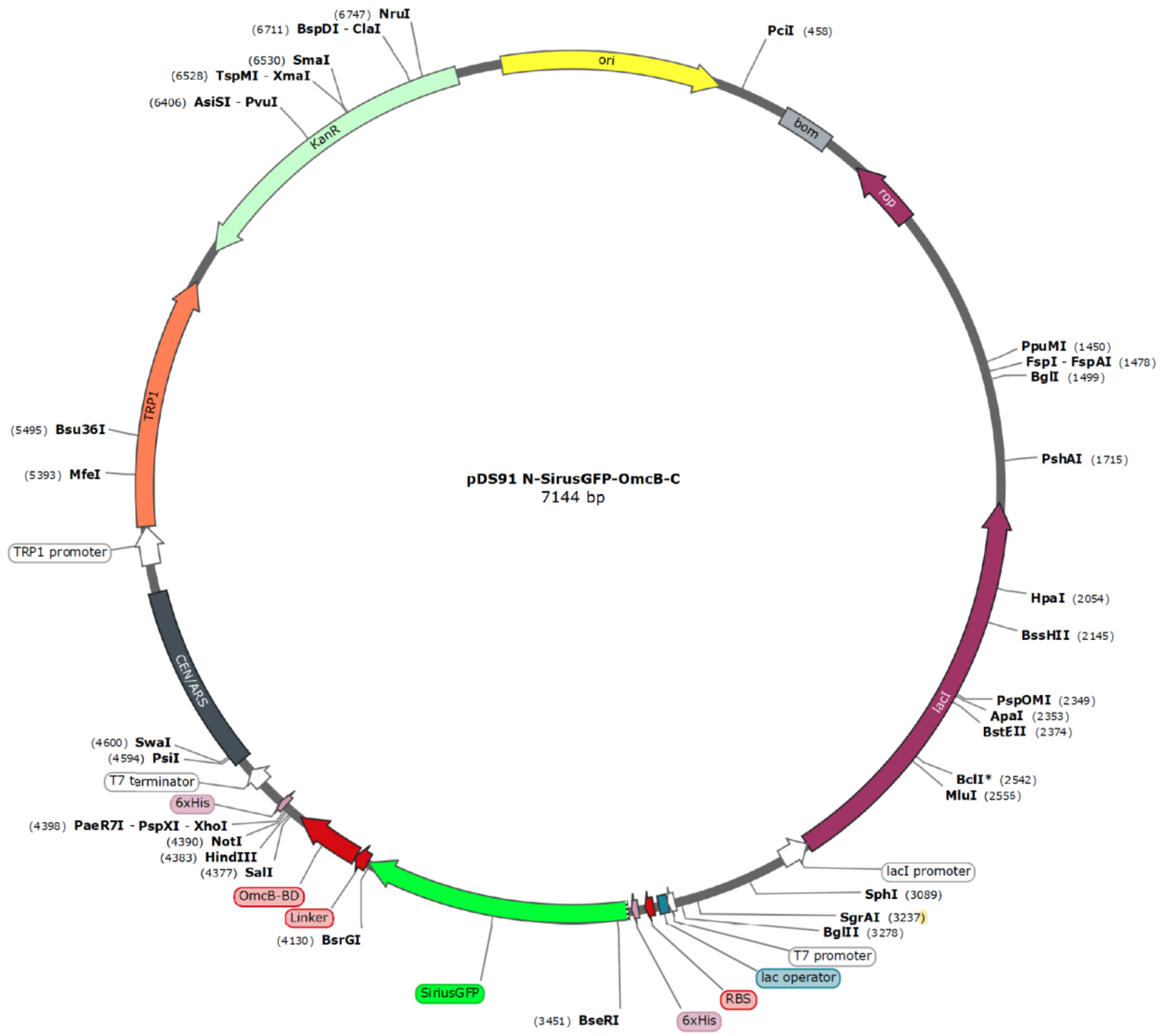

Figure S16. Plasmid pDS91 was used for the expression of GFP-OmcB-BD in *Escherichia coli*. pDS91 is a shuttle vector between *Escherichia coli* (*ori* – origin of replication, *KanR* – selection marker kanamycin) and *Saccharomyces cerevisiae* (*ARS* - origin of replication, *CEN* – centromeric DNA for mitosis, *TRP1* - selection marker tryptophan).

The complete GFP-OmcB-BD fusion protein consists of

- an N-terminal 6xHis affinity tag (for affinity purification; plasmid position bp 3397 – bp 3414);
- followed by the SiriusGFP tag (enhanced GFP derivative, robust folding and enhanced photostability, ref.: DOI: 10.1016/j.jneumeth.2018.12.008; bp 3424 - bp 4137);
- a 12 amino acid-long linker: GSAGSAAGSGEF, bp 4138 – bp 4173);
- OmcB-BD (bp 4174 – bp 4353).
